# Terpyridine–Cu(ii) targeting human telomeric DNA to produce highly stereospecific G-quadruplex DNA metalloenzyme†
†Electronic supplementary information (ESI) available: Experimental details, ESI figures and tables, ^1^H NMR spectra and HPLC traces. See DOI: 10.1039/c5sc01381j


**DOI:** 10.1039/c5sc01381j

**Published:** 2015-06-24

**Authors:** Yinghao Li, Mingpan Cheng, Jingya Hao, Changhao Wang, Guoqing Jia, Can Li

**Affiliations:** a State Key Laboratory of Catalysis , Dalian Institute of Chemical Physics , Chinese Academy of Sciences , Dalian 116023 , China . Email: canli@dicp.ac.cn ; Email: gqjia@dicp.ac.cn; b University of Chinese Academy of Sciences , No. 19A Yuquan Road , Beijing , 100049 , China

## Abstract

The cofactors commonly involved in natural enzymes have provided the inspiration for numerous advances in the creation of artificial metalloenzymes. Nevertheless, to design an appropriate cofactor for a given biomolecular scaffold or *vice versa* remains a challenge in developing efficient catalysts in biochemistry. Herein, we extend the idea of G-quadruplex-targeting anticancer drug design to construct a G-quadruplex DNA metalloenzyme. We found that a series of terpyridine–Cu(ii) complexes (CuL*n*) can serve as excellent cofactors to dock with human telemetric G-quadruplex DNA. The resulting G-quadruplex DNA metalloenzyme utilising CuL1 catalyzes an enantioselective Diels–Alder reaction with enantioselectivity of >99% enantiomeric excess and about 73-fold rate acceleration compared to CuL1 alone. The terpyridine–Cu(ii) complex cofactors demonstrate dual functions, both as an active site to perform catalysis and as a structural regulator to promote the folding of human telemetric G-quadruplex DNA towards excellent catalysts.

## Abstract 2

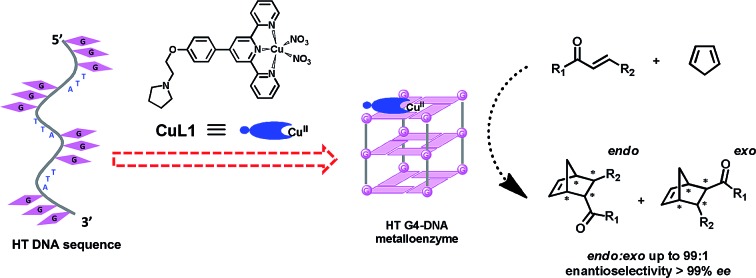
A highly stereospecific G-quadruplex DNA metalloenzyme was found by exploring the G-quadruplex targeting ligand pool.

## Introduction

Enzymes, the catalysts of biological systems, can mediate a wide range of chemical reactions with high catalytic rates and perfect regio-/stereoselectivity.^1^,^2^ Cofactors usually play a crucial role in this process.^1^ Metal complexes are one important type of cofactor that occur in many natural proteins to mediate various reactions.^3^ In a biomimetic spirit, catalytic metal complexes have also been widely used as cofactors to combine covalently or non-covalently with biomacromolecules, such as proteins, apo-enzymes and antibodies, to construct artificial metalloenzymes.^4^–^8^ In particular, the chiral feature of biomacromolecules makes artificial metalloenzymes an ideal catalyst for enantioselective reactions.^9^–^12^

DNA has served as a new biological scaffold in constructing artificial metalloenzymes for enantioselective catalysis.^12^–^14^ Roelfes, Boersma and Feringa found the cofactor Cu(ii)-(4,4′-dimethyl-2,2′-dipyridyl) could be most compatible with double-stranded DNA (dsDNA).^15^ The resulting artificial dsDNA metalloenzyme proceeds with excellent enantioselectivity in a series of enantioselective reactions.^16^–^26^ Recently, G-quadruplex DNA, due to its diverse and tunable chiral structures,^27^–^31^ has become another candidate in constructing DNA metalloenzymes for enantioselective reactions. Considerable efforts have been made to develop cofactors for G-quadruplex DNA, including a Cu(ii)–bipyridine complex and its analogs by Moses *et al.*,^32^ Cu(ii) ions by our group,^33^–^36^ and Cu(ii) cationic porphyrin by Hennecke *et al.*^37^ However, these G-quadruplex metalloenzymes only provide modest enantioselectivity for enantioselective reactions, which is most possibly due to the fact that these cofactors cannot fit well with the G-quadruplex DNA binding pocket, and consequently the resulting catalytic centers are not able to provide suitable interactions with prochiral substrates to promote efficient enantioselectivity. Therefore, to develop a suitable cofactor with the best fit to the binding pocket provided by G-quadruplex DNA is a key issue in constructing efficient artificial G-quadruplex metalloenzymes.

Through the lens of molecular biology, G-quadruplex DNAs are attractive targets for the development of anticancer drugs.^38^,^39^ Small molecules capable of inducing and stabilizing G-quadruplex DNA structure in human telomeres and oncogene-promoter regions have shown therapeutic intervention in cancer.^40^–^43^ It is reported that a series of terpyridine–metal complexes shows good affinity and high selectivity for human telomeric G-quadruplex DNA.^44^,^45^ Inspired by this specific recognition, in this work we have employed terpyridine–Cu(ii) complexes (CuL*n*) as cofactors to construct human telomeric G-quadruplex DNA metalloenzymes. Our study shows that the resulting human telomeric G-quadruplex DNA–CuL1 can afford excellent catalytic function in a Diels–Alder reaction with both high enantioselectivity over 99% enantiomeric excess (ee) and about 73-fold rate increase compared to CuL1 alone. To the best of our knowledge, this is the most efficient G-quadruplex DNA metalloenzyme for Diels–Alder reaction reported so far. Circular dichroism (CD) spectroscopic analysis, UV melting experiment and isothermal titration calorimetry (ITC) further indicate that the terpyridine–Cu(ii) cofactor not only functions as a catalytic center but also plays an important role in inducing and stabilizing human telemetric G-quadruplex DNA with a catalytically active three-dimensional structure. Based on this proof-of-concept experiment, it is envisioned that a much broader range of G-quadruplex metalloenzymes could be found by exploring the G-quadruplex-targeting ligand pool.

## Results

### Terpyridine–Cu(ii) complex targeting G-quadruplex DNA to produce artificial metalloenzyme

**Fig. 1:**
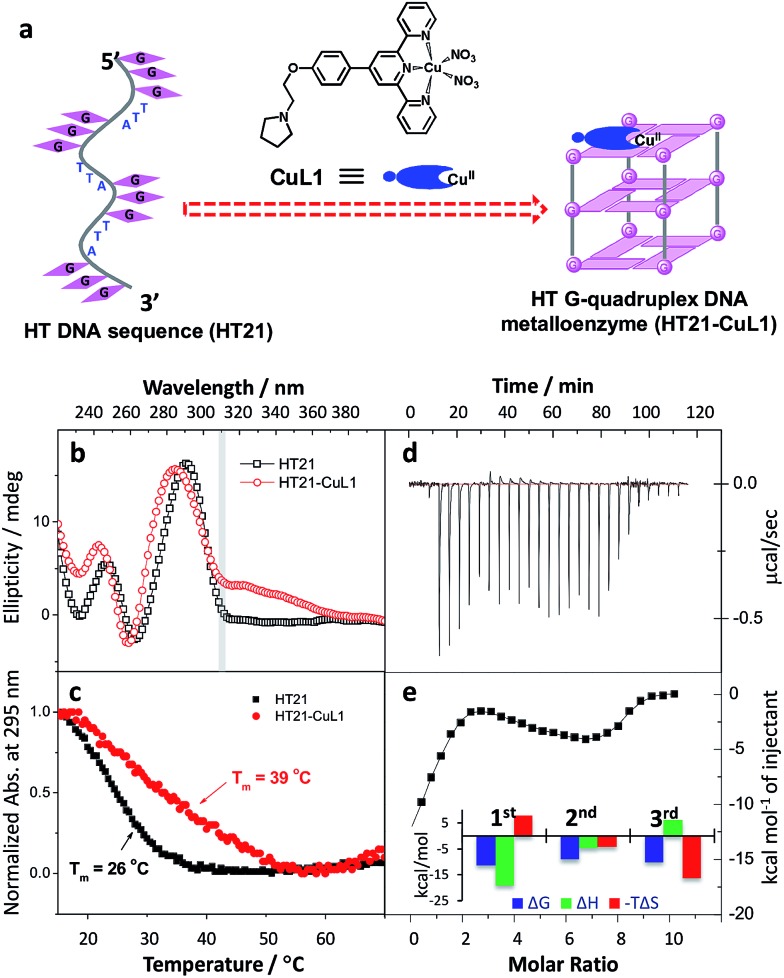
Construction of human telomeric G-quadruplex DNA metalloenzyme. (a) Schematic diagram of molecular recognition between terpyridine–Cu(ii) complex (CuL1) and human telomeric DNA sequence (HT21: 5′-(GGGTTA)_3_GGG-3′). (b) CD spectra. (c) UV melting curves monitored by UV absorption at 295 nm. (d and e) Isothermal titration calorimetry (ITC) experiment. Raw ITC data is shown in panel (d); plots of integrated calorimetric data after control subtraction (solid square), fitting curve (solid line) with a three-event binding model, and corresponding thermodynamic parameters are included in panel (e).

Inspired by the G-quadruplex DNA targeting nature of terpyridine–metal complexes, we synthesized the terpyridine–Cu(ii) complex (CuL1) and employed CuL1 to directly interact with a human telomeric DNA sequence (HT21) (Fig. 1a). CD spectroscopic analysis show that, upon interaction of CuL1 with HT21, an obvious spectral change in the region of 220–310 nm is observed, accompanied by a new CD band ranging from 310–370 nm (Fig. 1b). The spectral region of 310–370 nm originates from the electric transition of the terpyridine–Cu(ii) core.^46^ Therefore, the new CD band (310–370 nm) can be readily ascribed to the induced circular dichroism (ICD) signal of CuL1 imposed by the chiral surroundings of HT21, which is also direct evidence of the CuL1–HT21 association. The CD spectrum of 220–310 nm is usually indicative of the G-quadruplex DNA structure.^29^–^31^ However, it is complicated in this system by the overlapping absorption band of CuL1. To aid in understanding the CD results, UV melting experiments were performed (Fig. 1c). The melting temperature (*T*_m_) of HT21 increases by approximately 13 °C upon interaction with CuL1, which clearly indicates that the conformation of HT21 is significantly stabilized by the HT21–CuL1 complex formation. In this respect, the CD spectral change in the region of 220–310 nm contains much information on the structural transition of HT21. Moreover, the increased *T*_m_ clearly indicates that this structural transition occurs from a looser state to a more stable G-quadruplex DNA conformation. This deduction is further supported by the ITC experiment (Fig. 1d and e), which can determine the molecular nature of the binding interaction from a thermodynamic perspective.^47^ The binding isotherms reveal a complex binding process and can be best fit by a three-event model (Fig. 1e and Table S1†).^48^,^49^ The high-affinity binding site allows the interaction of one CuL1 molecule with HT21 by an affinity constant of approximately 10^8^ M^–1^. Thermodynamic parameters further show that this strong association process is driven by a highly favorable enthalpy and a less unfavorable entropic contribution. The favorable enthalpy can be linked to either Hoogsteen hydrogen bond formation or association between CuL1 and HT21, which contributes to the structural regulation of HT21 from a looser state to a more stable G-quadruplex DNA conformation, whereas the unfavorable entropy change is mainly ascribed to decreased freedom from both the compact G-quadruplex DNA structure and the bound state of CuL1.^50^

Upon confirming that CuL1 can directly induce and stabilize the HT21 G-quadruplex structure to form a stable HT21–CuL1 complex, we next selected the Diels–Alder reaction between azachalcone (**1a**) and cyclopentadiene (**2**) as a model to test the catalytic performance of HT21–CuL1. As shown in Table 1, HT21–CuL1 exhibits much higher catalytic performance than CuL1 alone (entry 2 *vs.* entry 1), and catalyzes the reaction with 92% conversion (turnover number (TON) = 9.2), high diastereoselectivity of product **3a** (*endo*/*exo* of 97 : 3) and 90% ee of the major *endo* isomer in 24 h (entry 2). Although these catalytic data were modest compared with the results with a dsDNA-based metalloenzyme,^15^ this result gives the best enantiomeric outcome concerning G-quadruplex DNA-based catalysis. This finding thus proves that CuL1 can act as an effective cofactor for the construction of a human telomeric G-quadruplex DNA metalloenzyme. More interestingly, combining the structural analysis and catalytic data, it is obvious that the CuL1 cofactor not only serves as a catalytic center but also shows structural regulation of human telomeric G-quadruplex DNA to promote excellent catalysis.

**Table 1:** Diels–Alder reaction catalyzed by human telomeric G-quadruplex DNA metalloenzyme

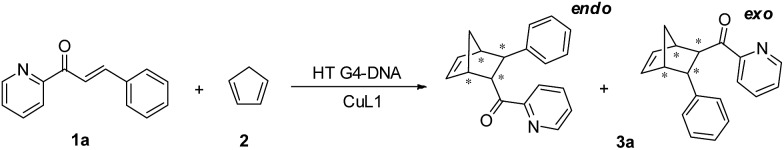					
Entry	DNA sequence (5′ → 3′)	M^+^	Conv.^*a*^ (%)	*endo*/*exo*^*b*^	ee^*b*^ (%, *endo*)
1^*c*^	—	—	42	71/29	0
2	HT21: (GGGTTA)_3_GGG	—	92	97/3	90
*Conformation stabilized by monovalent cations*					
3	HT21: (GGGTTA)_3_GGG	Na^+^	54	89/11	54
4		K^+^	48	88/12	49
5		NH_4_^+^	99	99/1	94
*Flanking base variants*					
6	A-HT21: *A*(GGGTTA)_3_GGG	NH_4_^+^	97	98/2	90
7	TA-HT21: *TA*(GGGTTA)_3_GGG	NH_4_^+^	96	98/2	94
8	TTA-HT21: *TTA*(GGGTTA)_3_GGG	NH_4_^+^	98	98/2	93
9	HT21-T: (GGGTTA)_3_GGG*T*	NH_4_^+^	97	97/3	88
10	HT21-TT: (GGGTTA)_3_GGG*TT*	NH_4_^+^	95	96/4	81
11	HT21-TTA: (GGGTTA)_3_GGG*TTA*	NH_4_^+^	95	96/4	73

^*a*^Determined for the crude product by HPLC analysis on a chiral stationary phase (ESI note 5), reproducible within ±2%.

^*b*^Determined by chiral-phase HPLC. Reproducible within ±2%.

^*c*^Cu(**L1**)(NO_3_)_2_ alone as catalyst. *Reaction conditions*: **1a** (1 mM), **2** (10 μL, 260 mM), human telomeric G-quadruplex DNA (50 μM), Cu(**L1**)(NO_3_)_2_ (100 μM), NaCl (50 mM) or KCl (150 mM) or NH_4_Cl (30 mM), MOPS buffer (0.5 mL, 20 mM, pH 6.5), 4 °C, 24 h.

### Structural optimization of G-quadruplex DNA in artificial metalloenzyme

In both natural and artificial metalloenzymes, subtle modifications of the secondary coordination sphere of the metal center usually exert a substantial influence on the catalytic function of the metalloenzyme.^12^,^51^ For the HT21–CuL1 metalloenzyme, this phenomenon provides an opportunity to improve the catalytic performance by varying the human telomeric G-quadruplex DNA structure. For this purpose, monovalent cations M^+^ (Na^+^/K^+^/NH_4_^+^) were introduced to induce HT21 to form a G-quadruplex structure in advance,^52^ and then CuL1 was added to construct an artificial human telomeric G-quadruplex DNA metalloenzyme, represented by HT21–M^+^–CuL1.

**Fig. 2:**
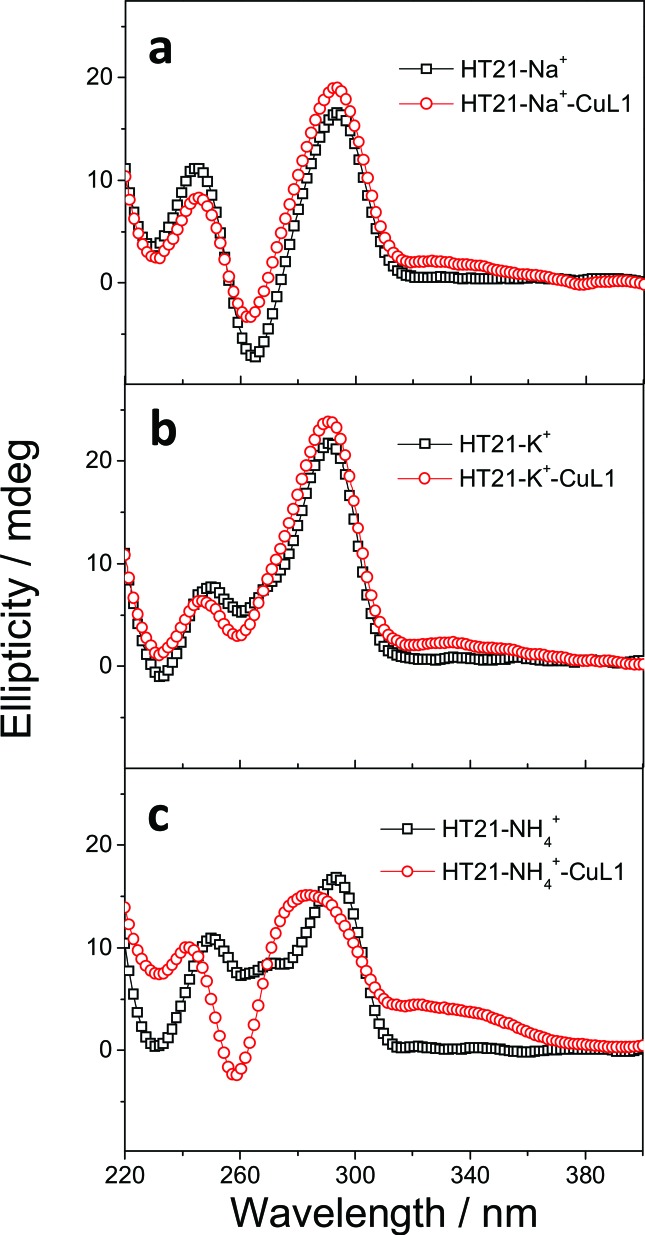
CD spectra of HT21 in the presence of different monovalent cations and their corresponding metalloenzyme. (a) 50 mM NaCl; (b) 150 mM KCl; (c) 30 mM NH_4_Cl (DNA strand concentration, 5 μM; CuL1, 10 μM; MOPS buffer, 20 mM, pH 6.5).

The CD spectra of HT21–Na^+^–CuL1 (Fig. 2a) and HT21–K^+^–CuL1 (Fig. 2b) indicate that CuL1 has less structural regulation for HT21 G-quadruplex DNA (220–310 nm), and the ICD signal for CuL1 (310–370 nm) is also obscure. However, for HT21–NH_4_^+^–CuL1, substantial structural change of HT21 G-quadruplex DNA (220–310 nm) and a clear ICD signal (310–370 nm) are observed (Fig. 2c). In addition, HT21–M^+^–CuL1 shows distinct catalytic behaviors for the Diels–Alder reaction compared with HT21–CuL1 (Table 1, entries 3–5 *vs.* entry 2). HT21–Na^+^–CuL1 shows an obvious decrease in both catalytic activity (conv. = 54%, TON = 5.4) and enantioselectivity (54% ee) (Table 1, entry 3). A similar case holds for HT21–K^+^–CuL1, with 48% conversion (TON = 4.8) and 49% ee (Table 1, entry 4). Surprisingly, when NH_4_^+^ was selected to stabilize HT21 G-quadruplex DNA, the HT21–NH_4_^+^–CuL1 metalloenzyme demonstrated a substantial increase in catalytic activity compared with HT21–CuL1, showing 99% conversion (TON = 9.9) and excellent diastereoselectivity (*endo*/*exo* of 99 : 1), and the enantioselectivity for the major *endo* product **3a** increased to 94% ee (Table 1, entry 5). For a comparison of intrinsic catalytic activity, we performed an enzymatic dynamics study. The Michaelis–Menten kinetics showed that the HT21–NH_4_^+^–CuL1 metalloenzyme reaches a catalytic efficiency (*k*_cat_/*K*_M_) of 36 ± 4 M^–1^ s^–1^ (*k*_cat_ = 0.0015 ± 0.0001 s^–1^, *K*_M_ = 41 ± 3 μM), which is approximately 73-fold more active than CuL1 (0.49 ± 0.06 M^–1^ s^–1^) (Fig. S1†). The rate acceleration in this system is comparable to dsDNA-based catalysis.^53^ At the same time, HT21–NH_4_^+^–CuL1 metalloenzyme shows 10-fold rate increase compared to HT21–CuL1 (Fig. S1†). Based on the above results, it is concluded that NH_4_^+^ ion can function as an efficient chaperone for the HT21 G-quadruplex DNA metalloenzyme to create a favorable modification of HT21 G-quadruplex DNA structure, which can help the CuL1 cofactor to fit well with the binding pocket within HT21 G-quadruplex DNA and consequently promote the Diels–Alder reaction.

Under optimized experimental conditions in NH_4_^+^ media, we further modified the HT21 G-quadruplex structure by introducing 1–3 flanking bases at the 5′ or 3′ end. These two positions were selected because the binding of the terpyridine–Cu(ii) complex with human telomeric G-quadruplex DNA suggests that the aromatic surface around the copper could interact with human telomeric G-quadruplex DNA by π–π stacking on the external G-quartet.^44^ Therefore, the flanking bases at the 5′ or 3′ end are also expected to modify the microenvironment of the CuL1 cofactor in the HT21–NH_4_^+^–CuL1 metalloenzyme. For the Diels–Alder reaction between **1a** and **2**, these derivative human telomeric G-quadruplex DNA metalloenzymes present an interesting effect on the enantioselectivity (Table 1, entries 6–11). The sequences with flanking bases at the 5′-end have little effect on enantioselectivity (90–94% ee) compared with HT21–NH_4_^+^–CuL1 (Table 1, entries 6–8 *vs.* entry 5). In contrast, a negative effect on enantioselectivity can be observed when the flanking bases are at the 3′-end (Table 1, entries 9–11 *vs.* entry 5). These data clearly indicate that the CuL1 cofactor is more sensitive to the 3′-end of HT21 G-quadruplex DNA, and a crowded microenvironment at the 3′-end is not favorable for high enantioselectivity.

**Fig. 3:**
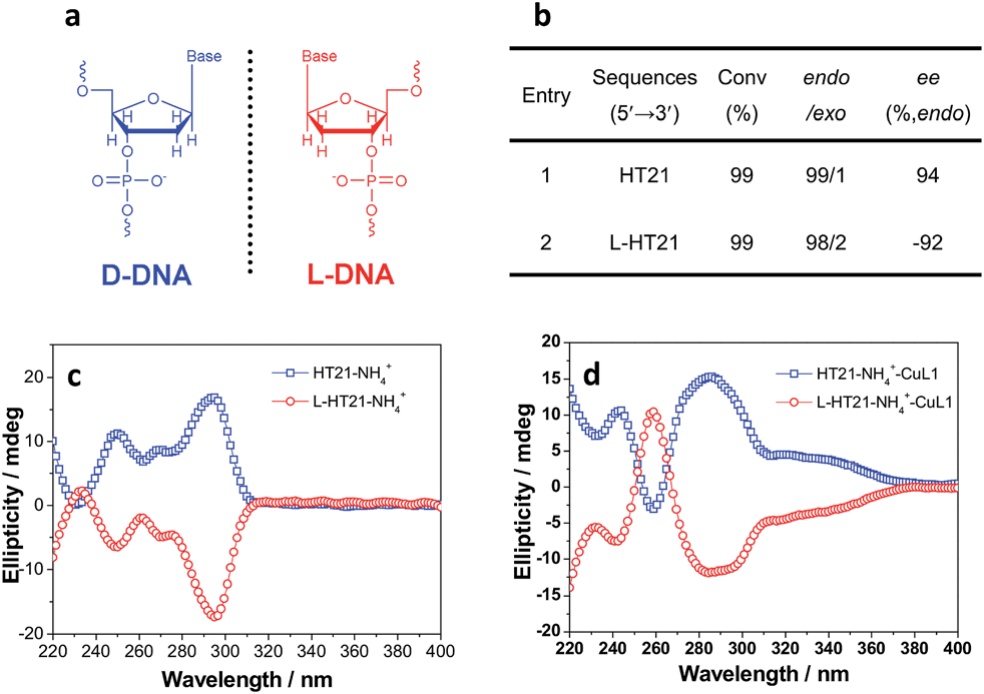
Inversion of global chirality of G-quadruplex DNA metalloenzyme. (a) Natural d-DNA building block for HT21 and its enantiomer for l-HT21. (b) The Diels–Alder reaction (**1a** and **2**) catalyzed by HT21 and l-HT21 G-quadruplex DNA metalloenzymes. (c) CD spectra of HT21 and l-HT21 in NH_4_^+^ media (30 mM NH_4_Cl). (d) CD spectra of HT21 G-quadruplex DNA metalloenzyme and l-HT21 G-quadruplex DNA metalloenzyme.

The above data strongly suggest that the conformation of HT21 G-quadruplex DNA plays a dominant role in the enantioselective control. Therefore, based on the high level of enantioselectivity afforded by HT21–NH_4_^+^–CuL1, we further considered whether we could selectively access either enantiomer in the Diels–Alder reaction by tuning the HT21 G-quadruplex DNA conformation. To this end, mutation was performed by replacing the naturally occurring d-DNA with l-DNA (Fig. 3a). CD spectra show that l-HT21 forms a G-quadruplex structure that is a mirror image of natural HT21 (Fig. 3c). More importantly, upon assembly with CuL1, the resulting l-HT21–NH_4_^+^–CuL1 metalloenzyme also presents the mirror image structure of natural HT21–NH_4_^+^–CuL1 (Fig. 3d). For the Diels–Alder reaction of **1a** and **2**, l-HT21–NH_4_^+^–CuL1 can successfully switch the absolute configuration of the major product **3a** from 94% ee to –92% ee (Fig. 3b).

### Structural optimization of cofactor structure in artificial metalloenzyme

Having validated the optimization of the second coordination sphere around Cu(ii) by tuning the G-quadruplex structures, we further optimized the CuL*n* cofactor by tuning the terpyridine ligand, namely the first coordination sphere around Cu^II^ (Table 2). It is found that high catalytic activity and high enantioselectivity are not specific to CuL1 as a cofactor but are also obtained for other terpyridine–Cu(ii) complexes possessing aminoethoxy side chains R (CuL*n*, *n* = 2–4) (Table 2, entries 1–4). In contrast, when the side chain (R) was changed from an aminoethoxy to a methyl group (CuL5), the HT21–NH_4_^+^–CuL5 metalloenzyme showed a substantial decline in both catalytic activity (conv. = 37%, TON = 3.7) and enantioselectivity (18% ee) (Table 2, entry 5). With further removal of the aromatic ring (CuL6), the HT21–NH_4_^+^–CuL6 metalloenzyme led to only 25% conversion (TON = 2.5) and a complete inhibition of enantioselectivity (Table 2, entry 6). These catalytic data show that both the aminoethoxy side chain and the aromatic ring are key components for the terpyridine–Cu(ii) cofactor to construct an efficient human telomeric G-quadruplex DNA metalloenzyme.

**Table 2:** The ligand effect of terpyridine–Cu(ii) cofactor on the catalytic function of human telomeric G-quadruplex DNA metalloenzyme

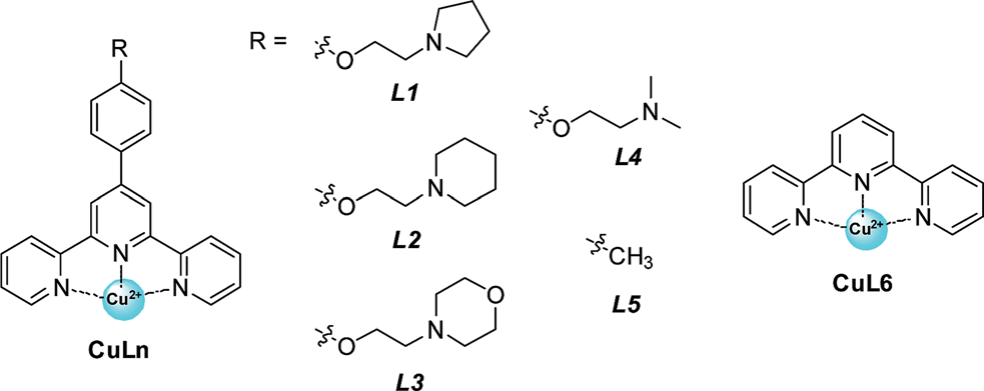				
Entry	Ligand	Conv.^*a*^ (%)	*endo*/*exo*^*b*^	ee^*b*^ (%, *endo*)
1	**L1**	99	99/1	94
2	**L2**	99	98/2	93
3	**L3**	99	97/3	92
4	**L4**	95	98/2	93
5	**L5**	37	81/19	18
6	**L6**	25	68/32	6

^*a*^Determined for the crude product by HPLC analysis on a chiral stationary phase (ESI note 5), reproducible within ±2%.

^*b*^Determined by chiral-phase HPLC. Reproducible within ±2%. *Reaction conditions*: **1a** (1 mM), **2** (10 μL, 260 mM), HT21 (50 μM), Cu(L*n*)(NO_3_)_2_ (100 μM), NH_4_Cl (30 mM), MOPS buffer (0.5 mL, 20 mM, pH 6.5), 4 °C, 24 h.

**Fig. 4:**
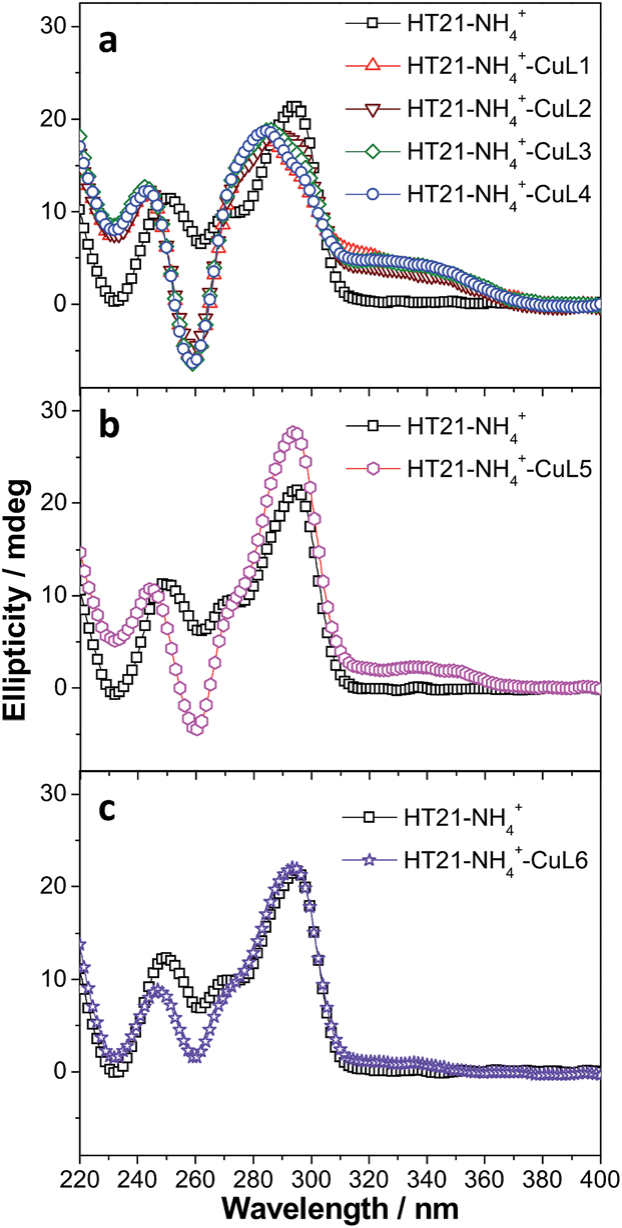
CD spectra of human telomeric G-quadruplex DNA metalloenzymes with different terpyridine–Cu(ii) cofactors compared with HT21 itself in NH_4_^+^ media. (a) CuL*n* (*n* = 1–4); (b) CuL5; (c) CuL6 (DNA strand concentration, 5 μM; NH_4_Cl, 30 mM; CuL*n*, 10 μM; MOPS buffer, 20 mM, pH 6.5).

To clarify the underlying reason, we further focused on structural understanding of the HT21–NH_4_^+^–CuL*n* metalloenzymes. We first used CD spectroscopy to detect the interaction of HT21–NH_4_^+^ with terpyridine–Cu(ii) complexes possessing different side chains (Fig. 4). In comparison with HT21–NH_4_^+^ itself, all test terpyridine–Cu(ii) cofactors demonstrate structural regulation on the HT21 G-quadruplex DNA structure (220–310 nm), but the degree of structural change is strongly dependent upon the type of side chain (R). At the same time, the ICD signals of the terpyridine–Cu(ii) cofactor (310–370 nm) imposed by HT21 G-quadruplex DNA also vary with different side chains. In detail, HT21–NH_4_^+^–CuL*n* (*n* = 1–4) metalloenzymes with terpyridine–Cu(ii) cofactors possessing aminoethoxy side chains show similar CD spectral features, with clear structural regulation of the HT21 G-quadruplex DNA (220–310 nm) accompanied by the appearance of a strong ICD signal of the CuL*n* cofactors (Fig. 4a). Upon changing the cofactor to CuL5 (Fig. 4b), the structural transition of HT21 G-quadruplex DNA is also observed but shows a distinct CD feature from the HT21–NH_4_^+^–CuL*n* (*n* = 1–4) metalloenzyme. Moreover, HT21–NH_4_^+^–CuL5 exhibits a smaller magnitude of ICD signal compared with HT21–NH_4_^+^–CuL*n* (*n* = 1–4). Upon changing to CuL6, the structural transition of HT21 becomes less notable, and the ICD signal is also obscured (Fig. 4c). The ICD signal in the wavelength region 310–370 nm originates from the electric transition of the terpyridine–Cu(ii) core, which is common to all six CuL*n* cofactors. Thus, the distinct amplitude of the ICD signal in HT21–NH_4_^+^–CuL*n* indicates a different molecular orientation of the terpyridine–Cu(ii) chromophore relative to the surrounding nucleobases,^54^ which gives direct evidence of different recognition between CuL*n* and HT21–NH_4_^+^ G-quadruplex DNA. More interestingly, a positive correlation between catalytic performance and amplitude of ICD signal indicates that the catalytic function of HT21–NH_4_^+^–CuL*n* metalloenzyme is strongly dependent on the molecular recognition between CuL*n* and HT21 G-quadruplex DNA.

This conclusion based on CD data is further supported by ITC data (Table 3, Fig. S2 and S3†). For terpyridine–Cu(ii) complexes possessing aminoethoxy side chains (CuL*n*, *n* = 1–4), all binding isotherms are similar and reveal obvious two-event binding process. The high-affinity binding process, with a binding constant (*K*_a1_) of 1.7 × 10^7^ M^–1^, is the result of a highly favorable entropy change and a smaller unfavorable enthalpy change. The low-affinity binding process displays a binding constant (*K*_a2_) of 5.8 × 10^5^–1.1 × 10^6^ M^–1^, which is 16–30 times lower than the *K*_a1_. In contrast to the high-affinity binding process, this low-affinity binding is favored by both entropy and enthalpy changes. The titration of CuL5 into HT21–NH_4_^+^ displays a relatively simple binding isotherm. A binding constant (*K*_a_) of 2.0 × 10^5^ M^–1^ is estimated by fitting a single binding event model, which is of the same order of magnitude as the low-affinity binding of CuL*n* (*n* = 1–4) to HT21–NH_4_^+^ G-quadruplex DNA. Furthermore, the molecular recognition process between CuL5 and HT21–NH_4_^+^ G-quadruplex DNA also shows a similar thermodynamic signature to the low-affinity binding of CuL*n* (*n* = 1–4) to HT21–NH_4_^+^ G-quadruplex DNA (Fig. S3†). In contrast, the binding isotherm of CuL6 to HT21–NH_4_^+^ is essentially the same as the background calorimetric titration of MOPS buffer to HT21–NH_4_^+^, suggesting that weak binding between CuL6 and HT21–NH_4_^+^ occurs, and neither the binding constant nor the thermodynamic parameters can be reliably determined. Thus, based on the different energetic characteristics, the ITC results clearly show three types of molecular recognition processes, and correspondingly, these three distinct interaction processes give the binding affinity order of CuL*n* (*n* = 1–4) > CuL5 > CuL6 to HT21–NH_4_^+^ G-quadruplex DNA. This sequence is consistent with not only the amplitude of the ICD signal in the CD experiment discussed above but also the Δ*T*_m_ values in UV melting experiments, where CuL*n* (*n* = 1–4) increases the melting temperature of HT21–NH_4_^+^ most significantly (Δ*T*_m_ ≈ 20 °C), followed by CuL5 (Δ*T*_m_ ≈ 10 °C) and CuL6 (Δ*T*_m_ ≈ 5 °C) (Fig. S4†).

**Table 3:** The binding thermodynamic parameters of different cofactors (CuL*n*) to human telomeric G-quadruplex DNA in 30 mM NH_4_Cl at 298 K

Cofactor	High-affinity binding site					Low-affinity binding site				
	*K*_a1_/M^–1^	*n*_1_	Δ*H*_1_^*a*^	–*T*Δ*S*_1_^*a*^	Δ*G*_1_^*a*^	*K*_a2_/M^–1^	*n*_2_	Δ*H*_2_^*a*^	–*T*Δ*S*_2_^*a*^	Δ*G*_2_^*a*^
CuL1^*b*^	(1.7 ± 0.2) × 10^7^	1.0	4.4 ± 0.1	–14.3	–9.9	(6.6 ± 0.5) × 10^5^	5.0	–2.2 ± 0.1	–5.7	–7.9
CuL2^*b*^	(1.7 ± 0.3) × 10^7^	1.1	4.2 ± 0.3	–14.0	–9.8	(1.0 ± 0.1) × 10^6^	5.8	–2.1 ± 0.1	–6.1	–8.2
CuL3^*b*^	(1.7 ± 0.3) × 10^7^	1.1	6.7 ± 0.2	–16.6	–9.9	(1.1 ± 0.1) × 10^6^	6.8	–1.5 ± 0.1	–6.8	–8.3
CuL4^*b*^	(1.7 ± 0.3) × 10^7^	1.2	4.2 ± 0.1	–14.1	–9.9	(5.8 ± 0.6) × 10^5^	5.4	–2.5 ± 0.1	–5.3	–7.8
CuL5^*c*^	(2.0 ± 0.5) × 10^5^	2.5	–1.1 ± 0.1	–6.1	–7.2	—	—	—	—	—
CuL6^*d*^	—	—	—	—	—	—	—	—	—	—

^*a*^Units are kcal mol^–1^.

^*b*^The data were obtained by the two-event binding model.

^*c*^The data were obtained by the one-event binding model.

^*d*^The affinity between the reactants is too low, neither the affinity nor the enthalpy of binding can be reliably determined.

In connection with the catalytic data and the above characterizations (CD, UV melting and ITC), we thus concluded that the molecular recognition between HT21 G-quadruplex DNA and terpyridine–Cu(ii) plays an essential role in the catalytic function of the HT21–NH_4_^+^–CuL*n* metalloenzyme. In fact, the high affinity of CuL*n* (*n* = 1–4) for HT21 G-quadruplex DNA can be easily understood as the interplay of two effects: (1) the π–π stacking interactions between the terpyridine and the G-quartet; (2) the electrostatic interaction between the positive charge of the aminoalkyl group and the negative charge of the phosphate backbones.^44^,^55^

### Substrate scope

**Fig. 5:**
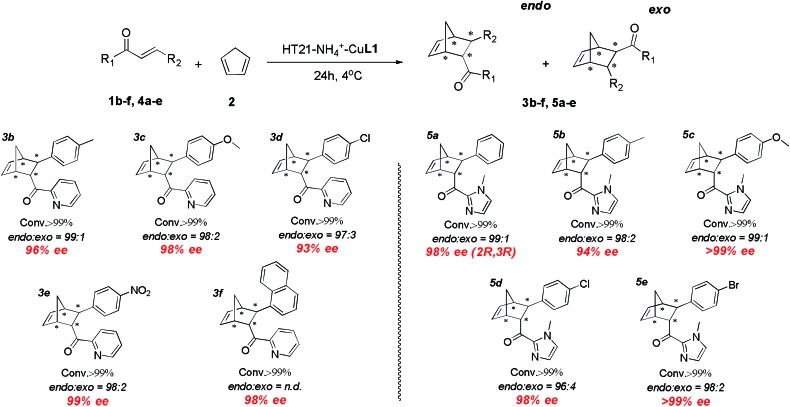
Substrate scope for human telomeric G-quadruplex DNA metalloenzyme. *Reaction conditions*: **1** or **4** (1 mM), 2 (10 μL, 260 mM), HT21 (50 μM), CuL1 (100 μM), NH_4_Cl (30 mM), MOPS buffer (20 mM, pH 6.5), 4 °C, 24 h. All data are averaged over two experiments and are reproducible within ±2%. Conversion was measured by ^1^H NMR analysis of the crude product. The *endo*/*exo* ratios and ee (for the *endo* isomer) were determined by chiral-phase HPLC. The absolute configuration of **5a** was obtained by literature comparison.^16^

With the excellent HT21–NH_4_^+^–CuL1 metalloenzyme in hand, we further tested the substrate specificity (Fig. 5). The HT21–NH_4_^+^–CuL1 metalloenzyme was found to be an active catalyst for all the tested aza-chalcone substrates (**1b–f**). In general, the introduction of a bulkier R_2_ group has a positive influence on the enantioselectivity. In particular, R_2_ with a nitro group afforded up to 99% ee (**3e**). Furthermore, high activity and enantioselectivity are also observed for the other type of dienophiles possessing acylimidazolyl groups (**4a–e**). Nearly pure isomers (>99% ee) are obtained for 4-MeO and 4-Br (**5c** and **5e**).

## Discussion

Small molecule targeting of G-quadruplex DNA represents an attractive approach to anticancer drug design.^40^–^43^ This recognition process is analogous to the assembly of an artificial enzyme between a catalytic cofactor and a biomolecular scaffold.^10^–^12^ Following this idea, we have found an excellent G-quadruplex metalloenzyme through molecular recognition between terpyridine–Cu(ii) and human telomeric G-quadruplex DNA, although the precise nature of the interaction is not well known. This modular assembly method allows rationally tunable enantioselectivity of the Diels–Alder reaction. The G-quadruplex DNA scaffold can be modulated by the addition of cation, modification of flanking bases at the 3′-end, and even inversion of global chirality by replacing natural d-DNA with unnatural l-DNA. Modification of the substituent in the terpyridine ligand can also change the binding affinity between G-quadruplex DNA and the terpyridine–Cu(ii) cofactor, which alters the microenvironment of the active site. Based on this proof-of-concept experiment, we expect this approach to provide a general method to find a broad variety of G-quadruplex metalloenzymes by screening G-quadruplex-targeting ligand pools.

## Conclusions

In conclusion, we have designed a G-quadruplex metalloenzyme that can afford both high catalytic activity and high enantioselectivity (up to 99% ee) for the Diels–Alder reaction. The success of this artificial metalloenzyme can be ascribed to the G-quadruplex DNA-targeting nature of terpyridine–Cu(ii). The terpyridine–Cu(ii) cofactor is found to play dual roles as both an active site for chemical catalysis and a structural regulator for the folding of the catalytic DNA structure. A positive correlation between the affinity of terpyridine–Cu(ii) for HT21 and the enantioselectivity of the Diels–Alder reaction is observed, which further confirms the importance of molecular recognition in constructing artificial G-quadruplex DNA metalloenzymes. Our work paves a new way to design G-quadruplex-based DNAzymes by learning from G-quadruplex-targeting drug design.

## Supplementary Material

Supplementary information
