# Supplementary material for: Terpyridine–Cu(ii) targeting human telomeric DNA to produce highly stereospecific G-quadruplex DNA metalloenzyme
Source: Chem Sci. 2015 Jun 24;6(10):5578–85. doi: 10.1039/c5sc01381j (PMC5949855; doi:10.1039/c5sc01381j)
Supplement: Supplementary file 1 [file SC-006-C5SC01381J-s001.pdf]

Electronic Supplementary Material (ESI) for Chemical Science.
This journal is © The Royal Society of Chemistry 2015

Supplementary Information

Terpyridine-Cu(II) Targeting Human Telomeric DNA to Produce Highly Stereospecific G-quadruplex DNA Metalloenzyme

Yinghao Li,[ab] Mingpan Cheng,[ab] Jingya Hao,[ab] Changhao Wang,[a] Guoqing Jia*[a] and Can Li*[a]

[a]State Key Laboratory of Catalysis, Dalian Institute of Chemical Physics, Chinese Academy of Sciences, Dalian 116023, China.

[b]University of Chinese Academy of Sciences, No.19A Yuquan Road, Beijing, 100049, China.

*E-mail: canli@dicp.ac.cn; gqjia@dicp.ac.cn.

Content

Supplementary Results 3
Supplementary Methods 12
General Methods.. 12
Supplementary Methods Note 1: Synthesis of substrates and racemic products 15
Supplementary Methods Note 2: Synthesis of ligands and metal complexes.. 15
Supplementary Methods Note 3: General catalytic procedure 19
Supplementary Methods Note 4: HPLC analysis condition 20
Supplementary Methods Note 5: Calculation the conversion of **1a** 20
Supplementary Methods Note 6: Kinetic measurements.. 21
^{1}H and ^{13}C NMR Spectra of Products.. 22
HPLC Traces of Products.. 37
References.. 60

Supplementary Results

Fig. S1 Catalytic activity of HT G4-DNA metalloenzymes for the Diels-Alder reaction of **1a** and **2** at pH 6.5. Smooth curve shows the fit to the Michaelis–Menten equation.

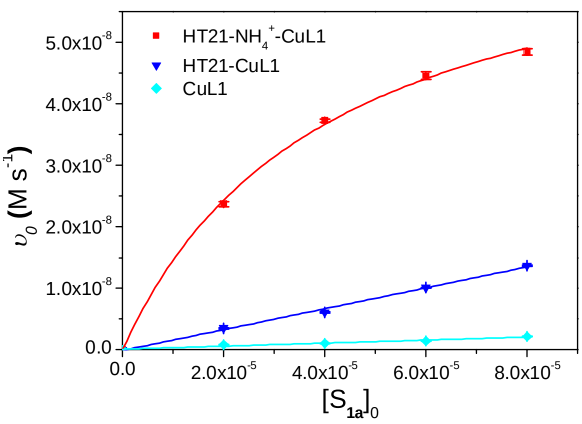

Entry	Catalyst	k_{cat}/K_M (M^{-1} s^{-1})[a]	K_M (μM)[a]	$k_{cat} \times 10^3$ (s^{-1})[a]	Rate acceleration (the ratio of k_{cat}/K_M)
1	CuL1	0.49 ± 0.06	N.D.	N.D.	**1**
2	HT21-CuL1	3.4±0.1	N.D.	N.D.	**~6.9**
3	HT21-NH_4^+-CuL1	36±4	41±3	1.5±0.1	**~73**

[a] Enzymatic kinetic parameters (k_{cat} and K_M) were obtained by fitting the kinetic data to the Michaelis–Menten equation ($\upsilon_0 = k_{cat}[E_{catalyst}]_0[S_{\mathbf{1a}}]_0/(K_M + [S_{\mathbf{1a}}]_0)$). k_{cat}/K_M values were obtained by fitting the linear portion of the Michaelis–Menten plot to $\upsilon_0 = (k_{cat}/K_M)[E_{catalyst}]_0[S_{\mathbf{1a}}]_0$. N.D., not determined.

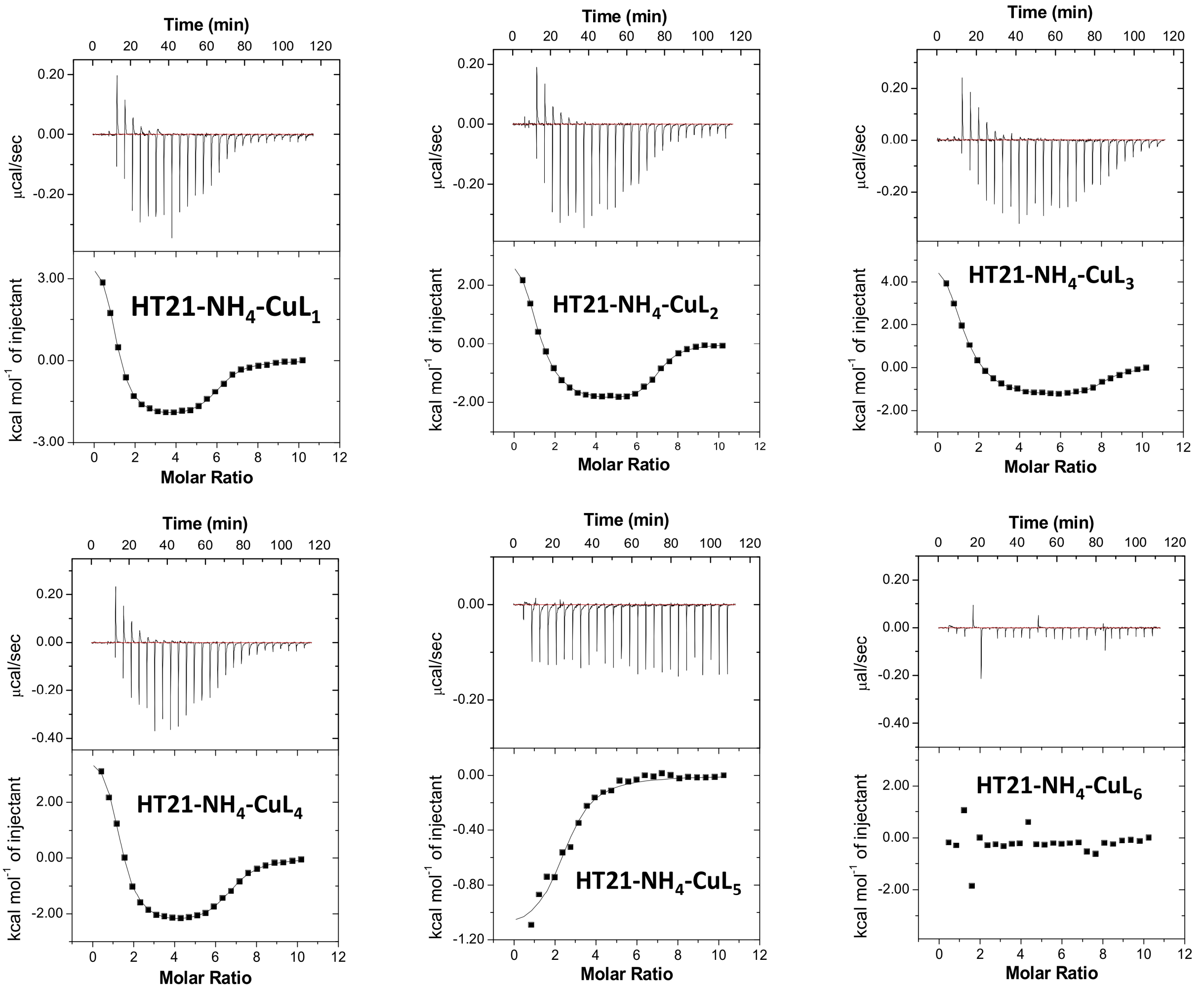

Fig. S2 Isothermal titration calorimetry (ITC) profile of different cofactors (CuLn) binding to human telomeric G-quadruplex DNA (HT21) in 30 mM NH_4Cl at 298K. Data were fitted to “two-event” (CuL1-L4) or “one-event” (CuL5) binding model using Origin 8.0 software.

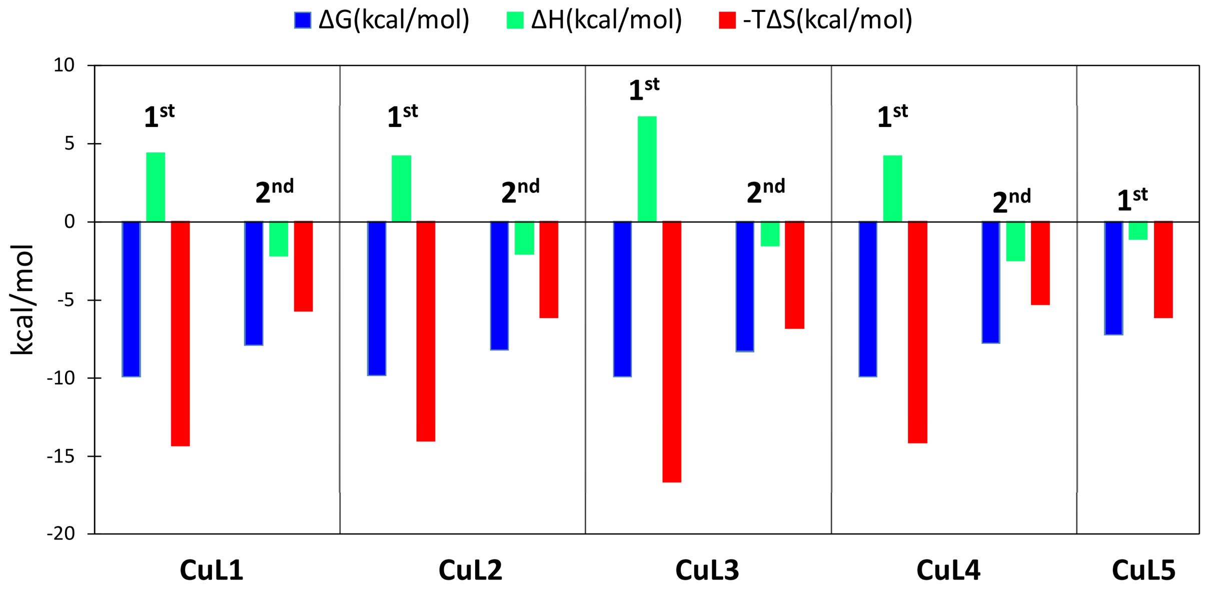

Fig. S3 Comparison of the thermodynamic signature of different cofactors (CuLn) binding to human telomeric G-quadruplex DNA (HT21) in 30 mM NH_4Cl at 298K.

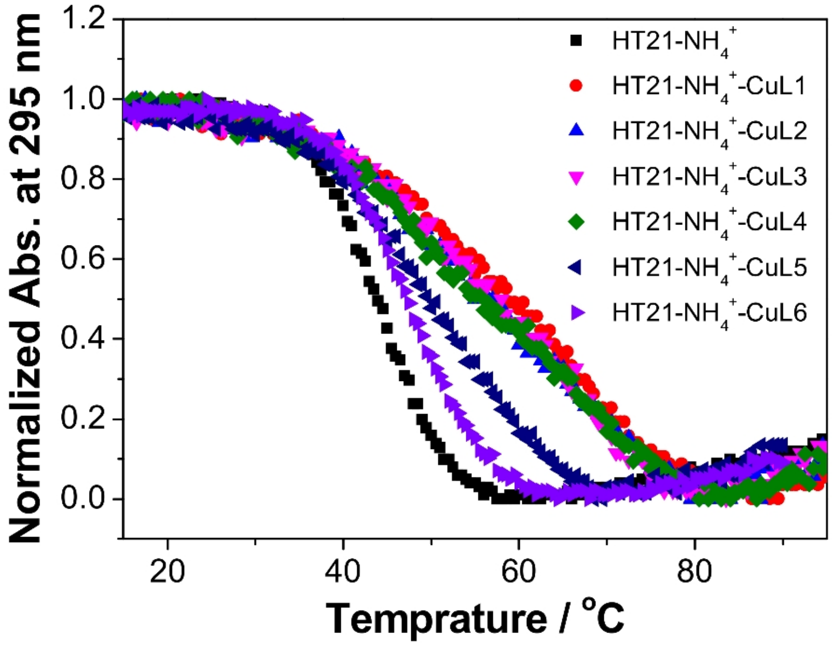

Fig. S4 Normalized melting curves of HT G4-DNA metalloenzymes with different terpyridine-Cu(II) cofactors as compared with HT21 itself in NH_4^+ media monitored by UV absorption at 295 nm (DNA strand concentration, 5 µM; NH_4Cl, 30 mM; CuLn, 10 µM; MOPS buffer, 20 mM, pH 6.5).

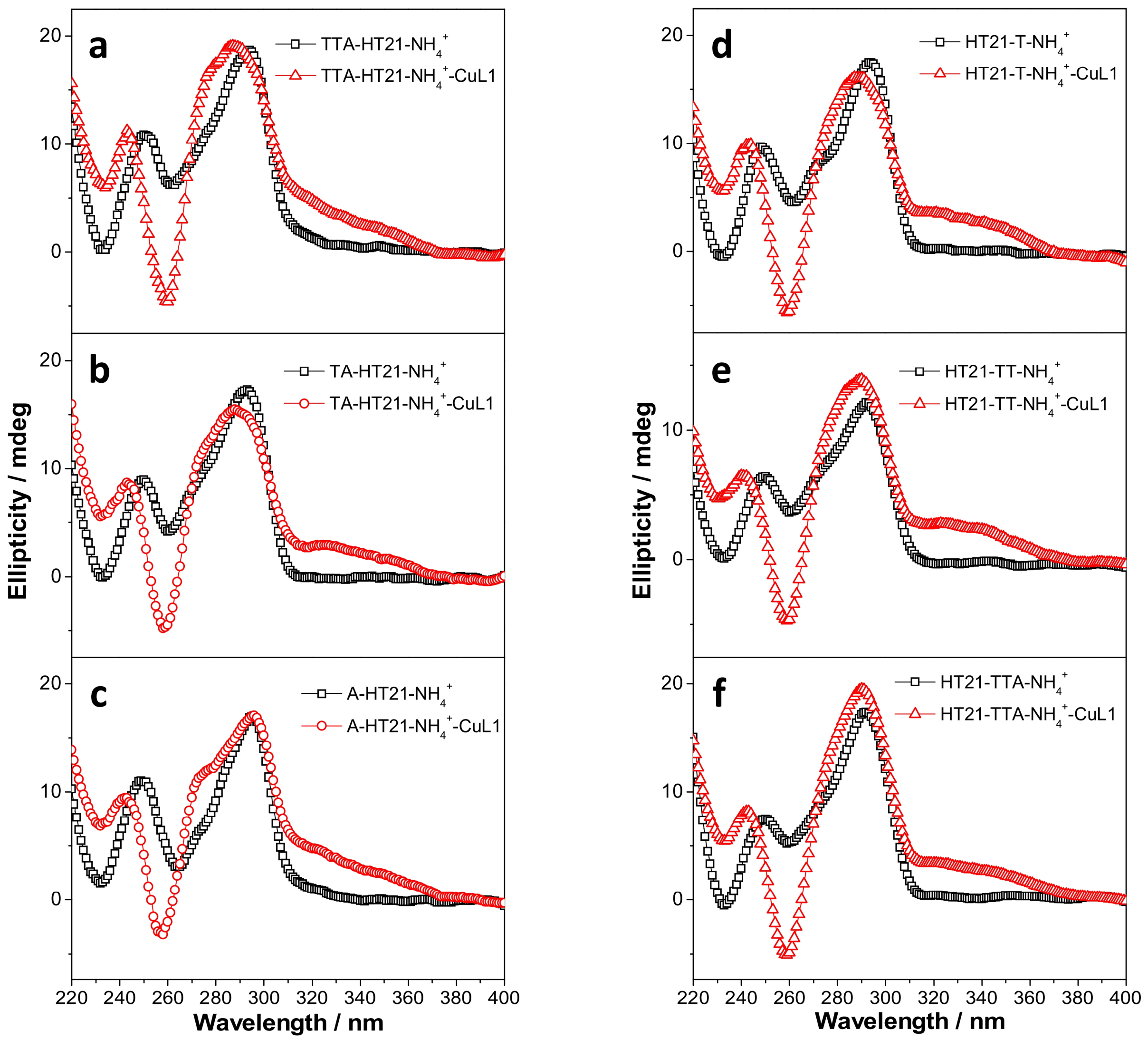

Fig. S5 CD spectra of derivative HT G4-DNA metalloenzymes with flanking bases at 5'-end (a-c) or 3'-end (d-f) (DNA strand concentration, 5 µM; NH_4Cl, 30 mM; CuL1, 10 µM; MOPS buffer, 20 mM, pH 6.5).

The stability of HT21-NH4$^+$-CuL1 is evaluated by recycling experiment (Fig. S6). After every reaction, the products were extracted with ether and fresh substrates were added into the aqueous phase containing HT21-NH_4^+-CuL1 catalyst. The results of recycling show that there are some degree of decrease in catalytic activity and enantioselectivity for G-quadruplex DNA metalloenzyme (Fig. S6a). This might be due to the structural change of G-quadruplex DNA metalloenzyme as indicated in CD spectra (Fig. S6b).

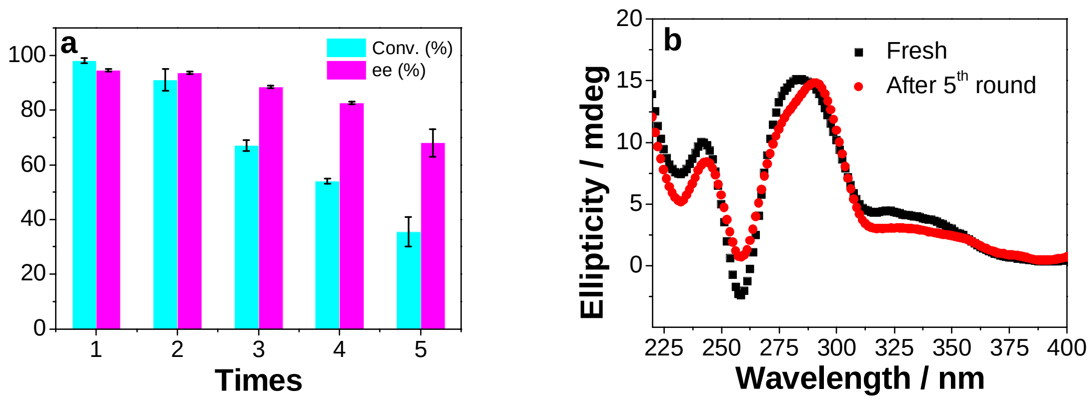

Fig. S6 Recycling experiments for evaluating the stability of HT21-NH_4^+-CuL1. (a) Catalytic results. (b) CD spectra of HT21-NH_4^+-CuL1 when fresh preparing or after 5th round catalysis.

We have used 2 equivalents of CuL1 compared to HT21 because the maximum enantiomeric excess (ee) can be obtained there.

As shown in Fig. S7a, the plot of the initial rate (v_0) as a function of [CuL1]:[HT21] shows that the activity increases dramatically until a molar ratio of 1:1 is reached, after which no significant increase can be observed. This indicates that a high active site has been formed at a molar ratio of 1:1.

However, a moderate enantioselectivity together with a big error bar is observed for the molar ratio of 1:1 (Fig. S7b). Until the ratio of [CuL1]:[HT21] increases to 2, the enantioselectivity reaches the maximum with negligible error bar. CD spectroscopic study further indicates that there is a great structural change of HT21 upon addition of 1 equivalent of CuL1 (Fig. S7c). Subsequently, a relatively stable structure is formed beginning with the molar ratio of 2. In combination with enantioselectivity and CD data, we deduce that the stable HT21 G-quadruplex DNA stabilized by 2 or more CuL1 is necessary for a highly specific enantioselectivity. In addition, ITC data tell us there are two binding events of CuL1 within HT21 G-quadruplex DNA, one is high-affinity and the other is low-affinity (Table 3). Therefore, we think that the first CuL1 with high-affinity is main active site and the second CuL1 with low-affinity mainly plays a structural role in constructing and stabilizing a catalytic 3D structure.

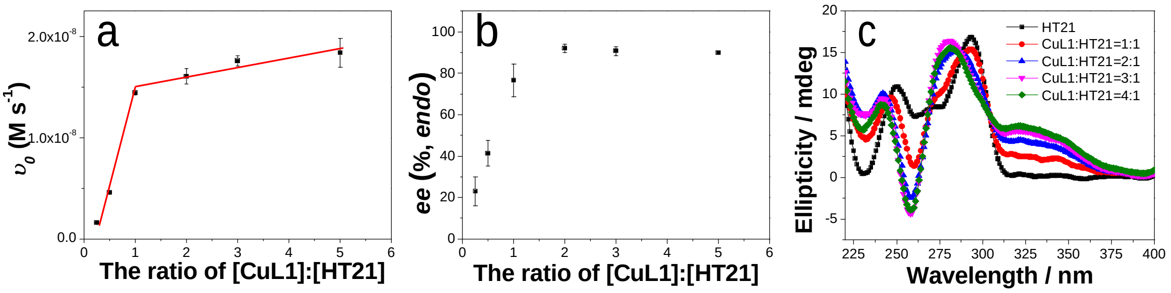

Fig. S7 Functional characterization of the catalysts. Plot of the initial rate (v_0) (a) and enantioselectivity (b) of model Diels-Alder reaction as a function of [CuL1]:[HT21] catalyzed by HT21-NH_4^+-CuL1. (c) CD spectra of different ratio of [CuL1]:[HT21] in 30 mM NH_4^+.

Table S1 Binding thermodynamic parameters of molecular recognition between terpyridine-Cu(II) complex (CuL1) and human telomeric DNA sequence (HT21) at 298K[a]

Binding events	K_a (M^{-1})	n	ΔH[b]	-TΔS[b]	ΔG[b]
1st	(1.5±0.6)E8	1.0	-19.1±3.1	7.9	-11.2
2nd	(3.2±0.9)E6	5	-4.7±0.3	-4.2	-8.9
3rd	(3.3±1.5)E7	2	6.2±1.5	-16.3	-10.1

[a] The data were obtained by the three sets of sites model.

[b] Units are kcal•mol^{-1}.

Table S2 Control experiments

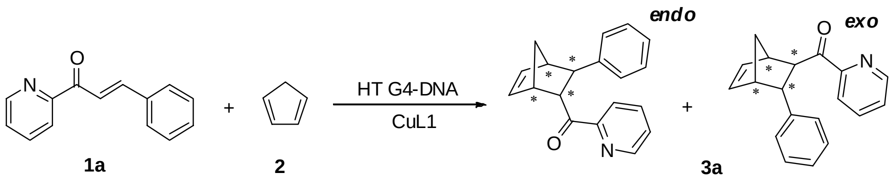

Entry	Catalyst	Conv.(%)[a]	*endo/exo*[b]	*ee* (%, *endo*)[b]
1	No	35	69/31	0
2	HT21-NH_4^+	46	70/30	17
3	HT21-NH_4^+-L1	32	66/34	0
4	HT21-NH_4^+-Cu^{2+}	>99	98/2	68

[a] Determined for the crude product by HPLC analysis on a chiral stationary phase (**Supplementary Methods Note 5**), Reproducible within ±5%. [b] Determined by chiral-phase HPLC. Reproducible within ±2%. Reaction conditions: **1a** (1 mM), **2** (10 uL, 260 mM), human telomeric G-quadruplex DNA (50 µM), **L1** (100 µM) or $Cu(NO_3)_2$ (100 µM), NH_4Cl (30 mM), MOPS buffer (20 mM, pH 6.5), 4 °C, 24h.

Supplementary Methods

General Methods

Materials: The 21-base human telomeric sequence 5′-d(GGGTTAGGGTTAGGGTTAGGG)-3′ (HT21) and its flanking base variants (see details in Table 1) were purchased from Sangon (Shanghai, China). Unnatural L-DNA was obtained from Takara (Dalian, China). The DNA strand concentrations were determined by measuring the UV absorbance of sample at 260 nm by using the molar extinction coefficient values provided by the manufacturer. $Cu(NO_3)_2{\cdot}3H_2O$ (>99.5%), NaCl (>99.5%), KCl (>99.5%), NH_4Cl (>99.5%) were purchased from the Shanghai Chemical Reagent Company of the Chinese Medicine Group. 3-(N-morpholino)propanesulfonic acid (MOPS) were purchased from Sangon (Shanghai, China). Water purified on a Milli-Q A10 water purification system (specific resistance of 18.2 MΩ at 25 °C) was used for all experiments. All experiments were carried out in 20 mM MOPS buffer (pH 6.5) unless otherwise stated. Ligands (L1-L4) and metal complexes CuLn (L1-L6) were synthesized as described in Supporting Information (see Supplementary Methods Note 2). L5 and L6 were obtained from commercial sources and used without further purification. Dienophiles **1a-f**, **4a-e** and their corresponding racemic products were prepared according to the literature (see **Supplementary Methods Note 1**).

UV Melting Experiment. UV melting experiments were carried out on a Shimadzu 2450 spectrophotometer (Shimadzu, Japan) equipped with a Peltier temperature control accessory. A sealed quartz cell with a path length of 1.0 cm was used. The UV melting curves of the human telomeric G-quadruplexes (5 μM) and their metalloenzymes were monitored by UV absorption at 295 nm with a heating rate of 0.5 °C/min. Data were analyzed by using Origin 8 software. The

melting temperatures (Tm) can be obtained from the best sigmoidal curve fit of the melting profile.

Circular Dichroism (CD) Spectroscopy. All CD spectra were recorded on a dual beam DSM 1000 CD spectrophotometer (Olis, Bogart, GA) with a 10 mm path-length quartz cell. Each measurement was recorded from 220 to 400 nm at 20 ºC under N_2 purge. The scan rate was 0.5 nm per second. The average scan for each sample was subtracted by a background CD spectrum of corresponding buffer solution. CD samples of all G-quadruplexes were prepared at a concentration (G-quadruplex unit) of 5 μM by using a MOPS buffer (20 mM, pH 6.5) containing corresponding salts (50 mM NaCl or 150 mM KCl or 30 mM NH_4Cl). When binding with CuLn complexes (10 μM), the CD spectra of corresponding human telomeric G-quadruplex DNA metalloenzymes were recorded then.

High Performance Liquid Chromatography (HPLC). The enantioselectivity was determined by Agilent HPLC 1260 analysis using Daicel chiralcel OD, ODH, OJH or chiralpak AD, ADH column with a UV-detector by using isopropanol and n-hexane as eluents at 25 °C.

^{1}H and ^{13}C NMR Spectroscopy. All ^{1}H and ^{13}C NMR spectra were recorded on a Bruker-400 MHz NMR instrument in $CDCl_3$ using TMS or residual protic solvent signals as internal standard. Data for ^{1}H NMR are recorded as follows: chemical shift (δ, ppm), multiplicity (s = singlet, d = doublet, t = triplet, q = quartet, m = multiplet or unresolved, coupling constant(s) in Hz, integration).

Isothermal Titration Calorimetry (ITC). ITC measurements were carried out at 25 °C using a MicroCal™ ITC_{200} titration calorimeter (MicroCal, GE). Experiments were performed in MOPS buffer (20 mM, pH 6.5) containing NH_4Cl (30 mM). The reference cell in the ITC was filled with ultrapure water (18.2 MΩ cm^{-1} resistivity). A pre-folded human telomeric G-quadruplex

DNA (20 μM) in NH_4Cl was loaded into the calorimeter cell. Then the syringe was loaded with CuLn complex (1 mM) in corresponding buffers. Following the auto-equilibration and an initial 500 s delay, the CuLn titrant divided into 25 injections (1.5 μL each) was added into the cell with 250 s injection intervals. The stir rate was 1000 rpm. All data were recorded with the GE Instruments software provided. Control experiments were conducted by titrating MOPS buffer into human telomeric G-quadruplex DNA and titrating CuLn into MOPS buffer under identical experimental condition to correct binding isotherm for background heat effects. Calorimetric data were further analyzed according to relevant model using MicroCal ORIGIN software and MATLAB. Data analysis gives ΔH (binding enthalpy change, kcal/mol), K_a (binding constant, M^{-1}), and n (number of bound CuLn cofactor) whereas the change in Gibbs energy and the entropic contribution were determined by the relationships $\Delta G = -RT \ln K_a$ and $\Delta G = \Delta H - T\Delta S$, respectively.

Supplementary Methods Note 1: Synthesis of substrates and racemic products

Dienophiles **1a**-**f**, **4a-e** and their corresponding racemic products were synthesized according to the literature.[1-5]

Supplementary Methods Note 2: Synthesis of ligands and metal complexes

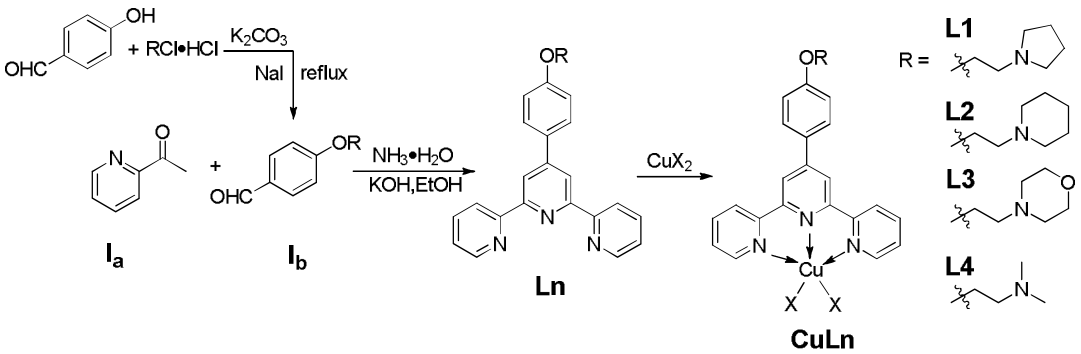

a) Synthesis of ligands (L1-L4).[6]

4'-substituted benzaldehyde (I_b): 4-hydroxybenzaldehyde (2.2 g, 18 mmol) and corresponding chloride hydrochloride (27 mmol) were dissolved in 100 mL of dried-acetone. Then, anhydrous potassium carbonate (4.97 g, 36 mmol) and sodium iodide (0.68 g, 4.5 mmol) was added to the flask. The reaction was refluxed under dried N_2 for about 24 hs. The flask was cooled to RT. The suspension is filtered, and the filtrate was concentrated *in vacuo*. The obtained solid was redissolved in EtOAc. The organic layer was washed with brine, and dried with Na_2SO_4. The drying agent was removed by filtration and the filtrate was concentrated and dried *in vacuo* to give a thick and pale yellow oil. The unpurified oil was used without further purification.

4’-substituted-2,2’:6’,2”-terpyridine (Ln): 2.61 mL of 2-acetylpyridine (2.813 g, 23.2 mmol, 2 equiv) was added to a solution of 4’-substituted benzaldehyde (11.6 mmol, 1 equiv) in 50 mL of ethanol. Then, KOH pellets (2.6 g, 85%, 46.5 mmol, 4 equiv) were added to this solution. The reaction was stirred at RT for 10 min. Subsequently, 40 mL of 25% aq. NH_3 was added to the flask drop-wise. After 24 h of stirring at 34 °C, 5 mL of 25% aq. NH_3 was added to the reaction mixture again. The flask was cooled to -20 °C and then the obtained white precipitate is filtered and washed with cool ethanol. We further purified the ligand by a subsequent recrystallization from ethanol-H_2O. The ligand was recovered by filtration, washed with cold ethanol and petroleum ether, and dried under high vacuum for 24 h.

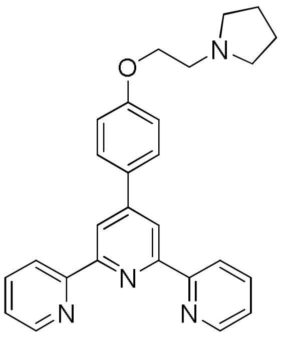

L1

4'-(4-(2-(pyrrolidin-1-yl)ethoxy)phenyl)-2,2':6',2''-terpyridine L1
White flaky crystal. **^{1}H NMR** (400 MHz, $CDCl_3$): δ (ppm) 8.75 – 8.69 (m, 4H), 8.66 (d, *J* = 8.0 Hz, 2H), 7.91 – 7.80 (m, 4H), 7.37 – 7.28 (m, 2H), 7.04 (d, *J* = 8.7 Hz, 2H), 4.18 (t, *J* = 5.9 Hz, 2H), 2.93 (t, *J* = 5.9 Hz, 2H), 2.70 – 2.57 (m, 4H), 1.89 – 1.74 (m, 4H). **^{13}C NMR** (101 MHz, $CDCl_3$): δ (ppm) 160.0, 156.5, 156.0, 149.9, 149.2, 136.9, 130.9, 128.6, 123.8, 121.5, 118.4, 115.1, 67.4, 55.2, 54.9, 23.7.

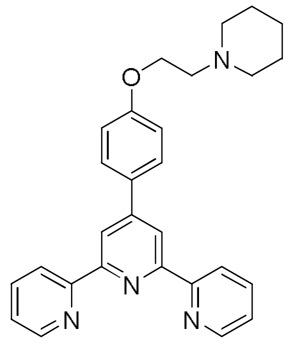

L2

4'-(4-(2-(piperidin-1-yl)ethoxy)phenyl)-2,2':6',2''-terpyridine (L2)
White flaky crystal. **^{1}H NMR** (400 MHz, $CDCl_3$): δ (ppm) 8.76 – 8.68 (m, 4H), 8.66 (d, *J* = 8.0 Hz, 2H), 7.91 – 7.81 (m, 4H), 7.37 – 7.29 (m, 2H), 7.03 (d, *J* = 8.7 Hz, 2H), 4.17 (t, *J* = 6.1 Hz, 2H), 2.80 (t, *J* = 6.1 Hz, 2H), 2.53 (t, 4H), 1.69 – 1.55 (m, 4H), 1.51 – 1.40 (m, 2H). **^{13}C NMR** (101 MHz, $CDCl_3$): δ (ppm) 159.9, 156.5, 155.9, 149.8, 149.2, 136.9, 130.8, 128.6, 123.8, 121.5, 118.4, 115.1, 66.3, 58.1, 55.2, 26.1, 24.3.

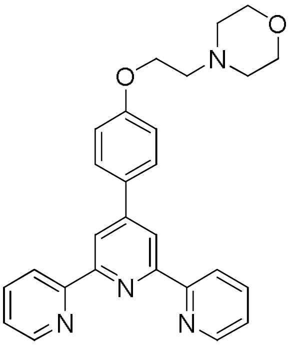

L3

4-(2-(4-([2,2':6',2''-terpyridin]-4'-yl)phenoxy)ethyl)morpholine (L3)

White needle-shaped crystal. **^{1}H NMR** (400 MHz, $CDCl_3$): δ (ppm) 8.75 – 8.69 (m, 4H), 8.66 (d, *J* = 7.9 Hz, 2H), 7.91 – 7.81 (m, 4H), 7.37 – 7.30 (m, 2H), 7.03 (d, *J* = 8.7 Hz, 2H), 4.17 (t, *J* = 5.7 Hz, 2H), 3.78 – 3.72 (m, 4H), 2.83 (t, *J* = 5.7 Hz, 2H), 2.64 – 2.55 (m, 4H). **^{13}C NMR** (101 MHz, $CDCl_3$): δ (ppm) 159.8, 156.5, 156.0, 149.8, 149.2, 137.0, 131.0, 128.6, 123.9, 121.5, 118.4, 115.1, 67.1, 66.1, 57.8, 54.3.

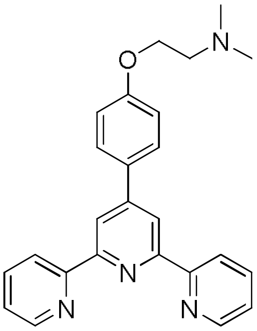

L4

2-(4-([2,2':6',2''-terpyridin]-4'-yl)phenoxy)-N,N-dimethylethanamine (L4)

White needle-shaped crystal. **^{1}H NMR** (400 MHz, $CDCl_3$): δ (ppm) 8.75 – 8.69 (m, 4H), 8.65 (d, *J* = 8.0 Hz, 2H), 7.91 – 7.81 (m, 4H), 7.36 – 7.29 (m, 2H), 7.04 (d, *J* = 8.6 Hz, 2H), 4.13 (td, *J* = 5.7, 0.8 Hz, 2H), 2.76 (t, *J* = 5.5 Hz, 2H), 2.36 (s, 6H). **^{13}C NMR** (101 MHz, $CDCl_3$): δ (ppm) 160.0, 156.6, 156.0, 149.9, 149.2, 137.0, 131.0, 128.6, 123.9, 121.5, 118.4, 115.1, 66.3, 58.5, 46.1.

b) Synthesis of metal complex CuLn (n = 1~6).[7]

4.8 mL of $Cu(NO_3)_2{\bullet}3H_2O$ (48.3 mg, 0.2 mmol, 1.0 equiv.) in anhydrous acetonitrile is added to a solution of 4′-substituted-2,2′:6′,2″-terpyridine (0.2 mmol, 1.0 equiv.) in 4.8 mL of anhydrous DCM and the solution is put in the fridge for 3-4 days. The obtained precipitate is filtered, washed with cool ethanol and dried under high vacuum for 24 h.

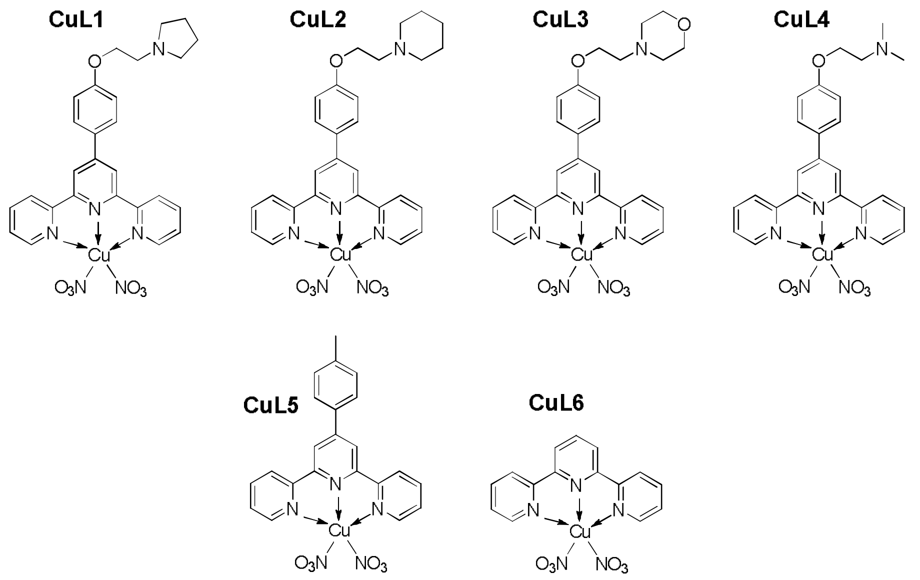

CuL1 is obtained as green solid. **HRMS** (TOF ES$^+$) calcd for $[C_{27}H_{26}ClN_4OCu]^+$: 520.1091, found: 520.1092.

CuL2 is obtained as green solid. **HRMS** (TOF ES$^+$) calcd for $[C_{28}H_{28}ClN_4OCu]^+$: 534.1248, found: 534.1254.

CuL3 is obtained as green solid. **HRMS** (TOF ES$^+$) calcd for $[C_{27}H_{26}ClN_4O_2Cu]^+$: 536.1040, found: 536.1049.

CuL4 is obtained as green solid. **HRMS** (TOF ES$^+$) calcd for $[C_{25}H_{24}ClN_4OCu]^+$: 494.0935, found: 494.0938.

CuL5 is obtained as blue crystal. **HRMS** (TOF ES$^+$) calcd for $[C_{22}H_{17}ClN_3Cu]^+$: 421.0407, found: 421.0410.

CuL6 is obtained as blue crystal. **HRMS** (TOF ES$^+$) calcd for $[C_{15}H_{11}ClN_3OCu]^+$: 330.9938, found: 330.9942.

Supplementary Methods Note 3: General catalytic procedure

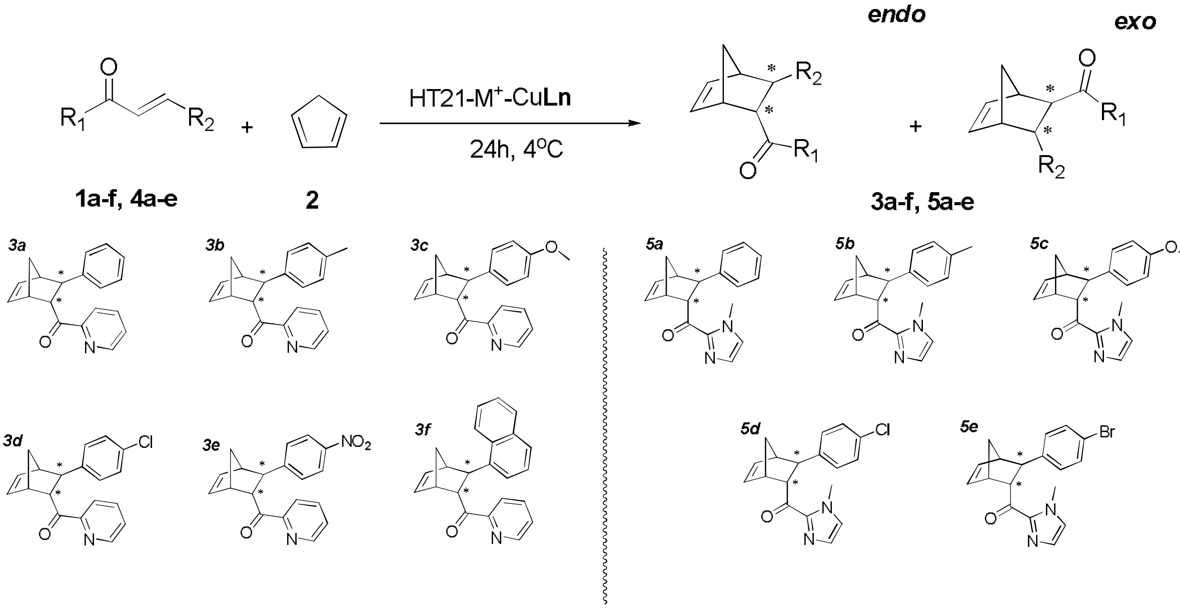

To a MOPS buffer (0.5 mL, 20 mM, pH 6.5) containing NaCl (50mM) or KCl (150 mM) or NH_4Cl (30 mM), an aqueous solution of human telomeric DNA (final G-quadruplex unit conc. 50 μM) was added. After stirred for a half hour at 4 °C, a solution of $Cu(Ln)(NO_3)_2$ (final conc. 100 μM) was added. Then, aza-chalcones **1** or **4** in CH_3CN (5 μL of a 0.1 M solution, 1 mM) was added. The reaction was initiated by the addition of freshly distilled cyclopentadiene **2** (10 μL, 260 mM) and the mixture was stirred for 24 hours at 4 °C, followed by the extraction with diethyl ether (3 × 5 mL), and the solvent was removed under reduced pressure. After a short flash chromatography, the residue was directly analyzed by ^{1}H-NMR and HPLC. The conversions were determined by ^{1}H-NMR and HPLC (only for **3a**) of the crude product.[8,9] The diastereoselectivity (*endo*/*exo*) and enantiomeric excess (*ee*) were determined by chiral HPLC.

Supplementary Methods Note 4: HPLC analysis condition

Product **3a**: Daicel Chiralcel-ODH, *n*-hexane/*i*-PrOH 98:2, flow rate 0.5 mL/min, λ = 212 nm).

Product **3b**: Daicel Chiralcel-ODH, *n*-hexane/*i*-PrOH 98:2, flow rate 0.5 mL/min, λ = 212 nm).

Product **3c**: Daicel Chiralpak-ADH, *n*-hexane/*i*-PrOH 95:5, flow rate 1.0 mL/min, λ = 212 nm).

Product **3d**: Daicel Chiralcel-OJH, *n*-hexane/*i*-PrOH 90:10, flow rate 0.5 mL/min, λ = 212 nm).

Product **3e**: Daicel Chiralpak-AD, *n*-hexane/*i*-PrOH 90:10, flow rate 1.0 mL/min, λ = 254 nm).

Product **3f**: Daicel Chiralpak-ADH, *n*-hexane/*i*-PrOH 90:10, flow rate 1.0 mL/min, λ =254 nm).

Product **5a**: Daicel Chiralcel-OD, *n*-hexane/*i*-PrOH 99:1, flow rate 0.5 mL/min, λ = 254 nm).

Product **5b**: Daicel Chiralpak-ADH, *n*-hexane/*i*-PrOH 97:3, flow rate 0.5 mL/min, λ = 254 nm).

Product **5c**: Daicel Chiralcel-OD, *n*-hexane/*i*-PrOH 98:2, flow rate 1.0 mL/min, λ = 254 nm).

Product **5d**: Daicel Chiralpak-AD, *n*-hexane/*i*-PrOH 85:15, flow rate 1.0 mL/min, λ = 254 nm).

Product **5e**: Daicel Chiralpak-ADH, *n*-hexane/*i*-PrOH 97:3, flow rate 1.0 mL/min, λ = 254 nm).

Supplementary Methods Note 5: Calculation the conversion of 1a

The procedure to determine the conversion of **1a** by HPLC was according to the literature.[8,9]

Conversions of **1a** were calculated using the formula:

Conversion (%) = $A_{3a} / (A_{3a} + A_{1a} / f)$

Where A_{1a} and A_{3a} are the HPLC peak areas of **1a** and **3a**, respectively. And f is the correction factor determined to be 0.73 from a calibration curve.

Supplementary Methods Note 6: Kinetic measurements

All kinetic measurements were performed using UV-Vis spectroscopy (Shimadzu 2450) at 293 K by monitoring the disappearance of the absorption of **1a** at 326 nm. Typical procedure is described as follows: G4-DNA (final G-quadruplex unit conc. 25 μM) was added to MOPS (20 mM, pH 6.5) containing NH_4Cl (30 mM) in a quartz cuvette. After stirring for 15 min, CuL1 complex (final conc. 50 μM) was added. After stirring for another 15 min, a series of concentration of [**1a**] equal to 20, 40, 60 and 80 μM was added. The determination was made after **2** (final conc. 16 mM) was added with the cuvette sealed tightly. The D-A reaction was regarded as pseudo-first-order reaction as **2** is present in excess and **1a** is present at low concentrations. Enzymatic kinetic parameters (k_{cat} and K_M) were obtained by fitting the data to the Michaelis–Menten equation ($\upsilon_0 = k_{cat}[E_{catalyst}]_0[S_{\mathbf{1a}}]_0/(K_M+[S_{\mathbf{1a}}]_0)$). k_{cat}/K_M values were obtained by fitting the initial linear portion of the Michaelis–Menten plot to $\upsilon_0 = (k_{cat}/K_M)[E_{catalyst}]_0[S_{\mathbf{1a}}]_0$.

^{1}H and ^{13}C NMR Spectra of Products

(3-phenylbicyclo[2.2.1]hept-5-en-2-yl)(pyridin-2-yl)methanone (**3a**)

^{1}H NMR (400 MHz, $CDCl_3$, *endo* isomer): δ 8.67 (m, 1H), 8.00 (m, 1H), 7.81 (m, 1H), 7.49 – 7.39 (m, 1H), 7.36 – 7.23 (m, 4H), 7.17 (m, 1H), 6.49 (m, 1H), 5.82 (m, 1H), 4.53 (m, 1H), 3.54 (s, 1H), 3.45 (d, *J* = 3.9 Hz, 1H), 3.09 (s, 1H), 2.07 (d, *J* = 8.4 Hz, 1H), 1.62 (m, 1H).

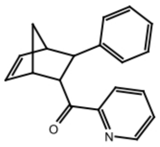

3a

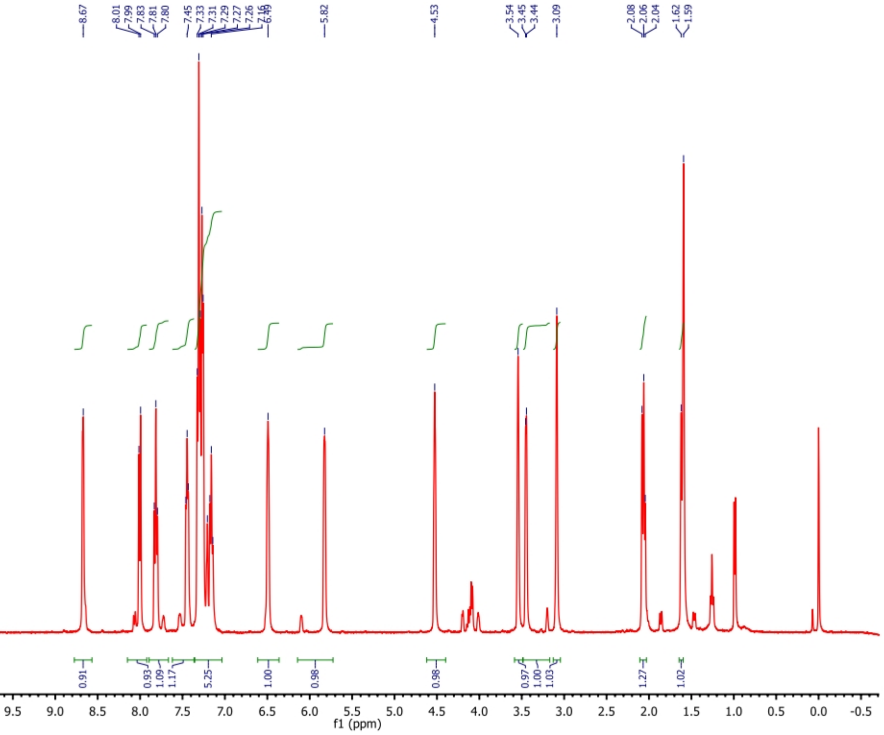

pyridin-2-yl(3-p-tolylbicyclo[2.2.1]hept-5-en-2-yl)methanone (**3b)**

^{1}H NMR (400 MHz, $CDCl_3$, *endo* isomer): δ 8.67 (d, *J* = 4.6 Hz, 1H), 8.00 (d, *J* = 7.8 Hz, 1H), 7.81 (td, *J* = 7.7, 1.3 Hz, 1H), 7.44 (dd, *J* = 7.0, 5.3 Hz, 1H), 7.21 (d, *J* = 8.0 Hz, 2H), 7.08 (d, *J* = 7.9 Hz, 2H), 6.49 (dd, *J* = 5.4, 3.3 Hz, 1H), 5.81 (dd, *J* = 5.6, 2.7 Hz, 1H), 4.52 (dd, *J* = 4.9, 3.7 Hz, 1H), 3.53 (s, 1H), 3.41 (d, *J* = 5.0 Hz, 1H), 3.05 (s, 1H), 2.30 (s, 3H), 2.06 (d, *J* = 8.4 Hz, 1H), 1.59 (dd, *J* = 8.5, 1.1 Hz, 1H).

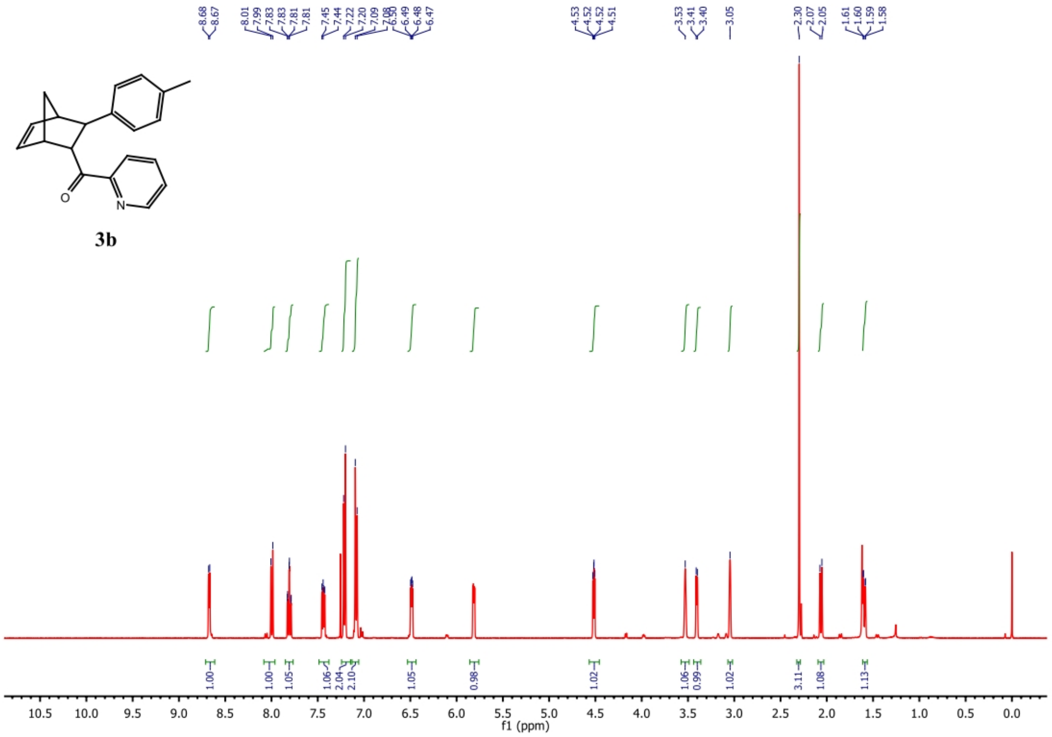

(3-(4-methoxyphenyl)bicyclo[2.2.1]hept-5-en-2-yl)(pyridin-2-yl)methanone (**3c)**

^{1}H NMR (400 MHz, $CDCl_3$, *endo* isomer): δ 8.74 – 8.60 (m, 1H), 8.00 (d, *J* = 7.9 Hz, 1H), 7.81 (td, *J* = 7.7, 1.7 Hz, 1H), 7.44 (ddd, *J* = 7.5, 4.8, 1.1 Hz, 1H), 7.28 – 7.18 (m, 2H), 6.86 – 6.78 (m, 2H), 6.48 (dd, *J* = 5.5, 3.2 Hz, 1H), 5.81 (dd, *J* = 5.6, 2.7 Hz, 1H), 4.49 (dd, *J* = 5.1, 3.5 Hz, 1H), 3.77 (s, 3H), 3.53 (s, 1H), 3.39 (d, *J* = 4.5 Hz, 1H), 3.02 (d, *J* = 1.2 Hz, 1H), 2.06 (d, *J* = 8.4 Hz, 1H), 1.60 (dd, *J* = 8.5, 1.6 Hz, 1H).

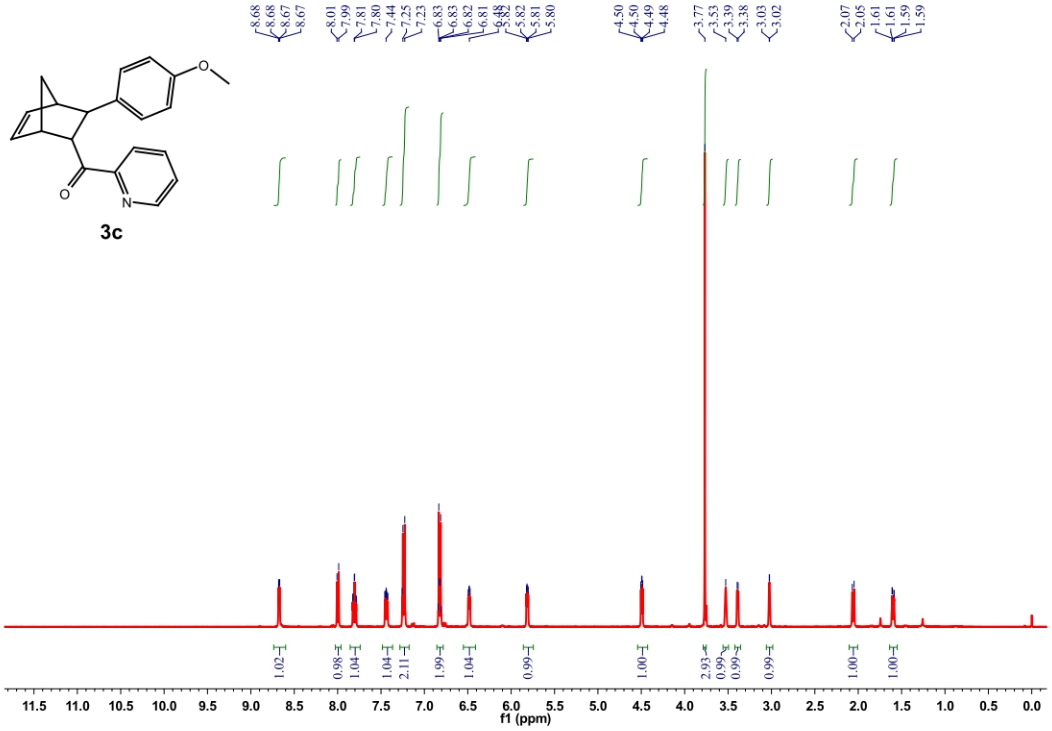

(3-(4-chlorophenyl)bicyclo[2.2.1]hept-5-en-2-yl)(pyridin-2-yl)methanone **(3d)**

^{1}H NMR (400 MHz, $CDCl_3$, *endo* isomer): δ 8.67 (d, *J* = 4.6 Hz, 1H), 8.00 (d, *J* = 7.8 Hz, 1H), 7.83 (dd, *J* = 8.5, 6.9 Hz, 1H), 7.46 (dd, *J* = 7.4, 4.9 Hz, 1H), 7.25 – 7.10 (m, 4H), 6.48 (dd, *J* = 5.4, 3.2 Hz, 1H), 5.83 (dd, *J* = 5.5, 2.7 Hz, 1H), 4.46 (dd, *J* = 5.1, 3.5 Hz, 1H), 3.54 (s, 1H), 3.40 (d, *J* = 5.1 Hz, 1H), 3.05 (s, 1H), 2.01 (d, *J* = 8.5 Hz, 1H), 1.62 (d, *J* = 8.6 Hz, 1H).

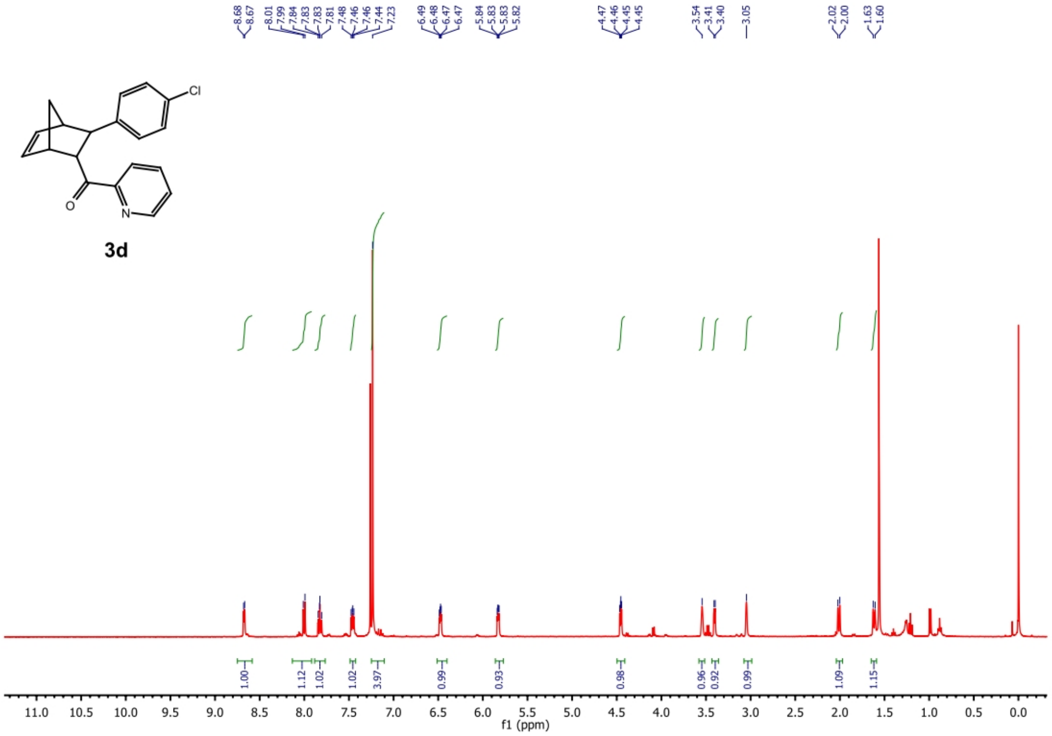

3d

(3-(4-nitrophenyl)bicyclo[2.2.1]hept-5-en-2-yl)(pyridin-2-yl)methanone **(3e)**

^{1}H NMR (400 MHz, *endo* isomer): δ 8.66 (d, *J* = 4.5 Hz, 1H), 8.07 (m, 3H), 7.84 (t, *J* = 7.6 Hz, 1H), 7.47 (m, 3H), 6.52 – 6.44 (m, 1H), 5.88 (dd, *J* = 5.3, 2.5 Hz, 1H), 4.51 – 4.44 (m, 1H), 3.66 – 3.50 (m, 2H), 3.13 (s, 1H), 2.00 (d, *J* = 8.6 Hz, 1H), 1.67 (d, *J* = 8.4 Hz, 1H).

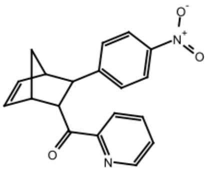

3e

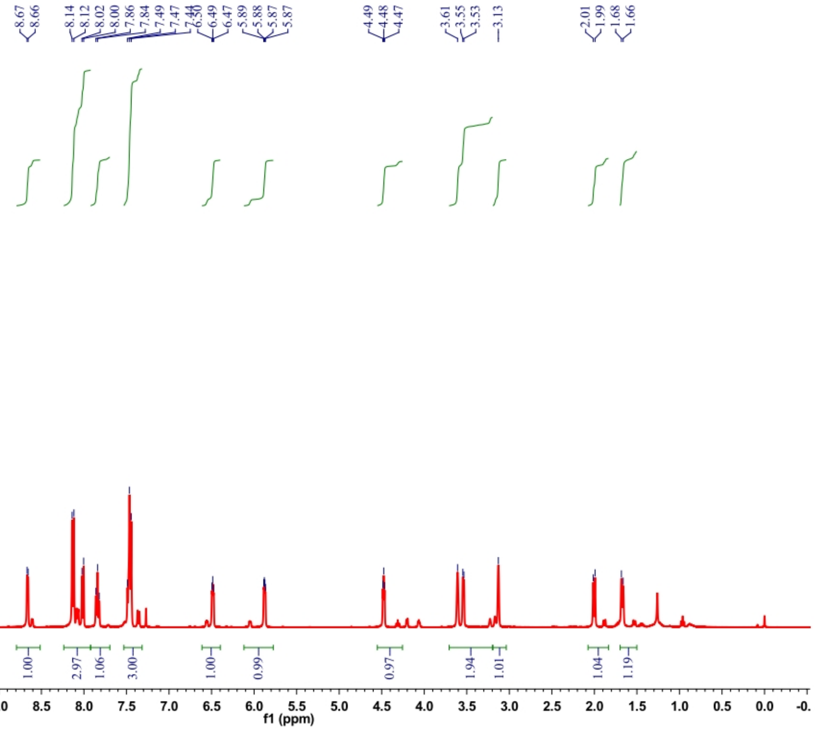

(3-(naphthalen-1-yl)bicyclo[2.2.1]hept-5-en-2-yl)(pyridin-2-yl)methanone (**3f**)

^{1}H NMR (400 MHz, $CDCl_3$, *endo* isomer): δ 8.70 – 8.57 (m, 1H), 8.18 – 7.98 (m, 2H), 7.87 – 7.76 (m, 2H), 7.70 (d, *J* = 8.2 Hz, 1H), 7.62 (d, *J* = 7.2 Hz, 1H), 7.49 – 7.37 (m, 4H), 6.68 – 6.57 (m, 1H), 5.97 – 5.89 (m, 1H), 4.74 – 4.65 (m, 1H), 4.07 (d, *J* = 4.8 Hz, 1H), 3.53 (s, 1H), 3.21 (s, 1H), 2.19 (dd, *J* = 8.4 Hz, 1H), 1.69 (dd, *J* = 8.3 Hz, 1H).

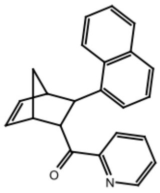

3f

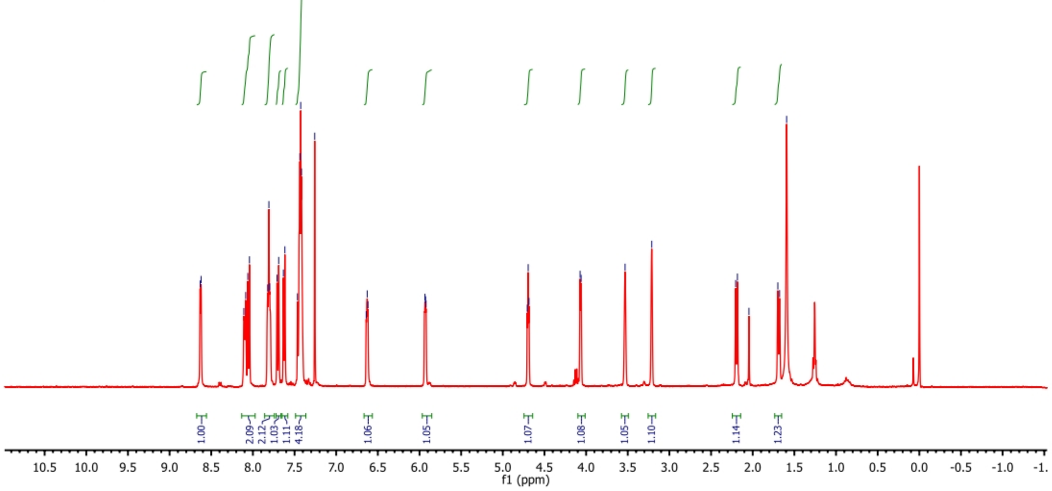

(1-methyl-1H-imidazol-2-yl)(3-phenylbicyclo[2.2.1]hept-5-en-2-yl)methanone (**5a**)

^{1}H NMR (400 MHz, $CDCl_3$, *endo* isomer): δ 7.26 – 7.15 (m, 4H), 7.11 – 7.03 (m, 2H), 6.93 (s, 1H), 6.44 (dd, *J* = 5.0, 3.4 Hz, 1H), 5.82 (dd, *J* = 5.4, 2.6 Hz, 1H), 4.35 – 4.26 (m, 1H), 3.89 (s, 3H), 3.53 (s, 1H), 3.28 (d, *J* = 5.1 Hz, 1H), 2.97 (s, 1H), 1.96 (d, *J* = 8.5 Hz, 1H), 1.52 (d, *J* = 8.5 Hz, 1H).

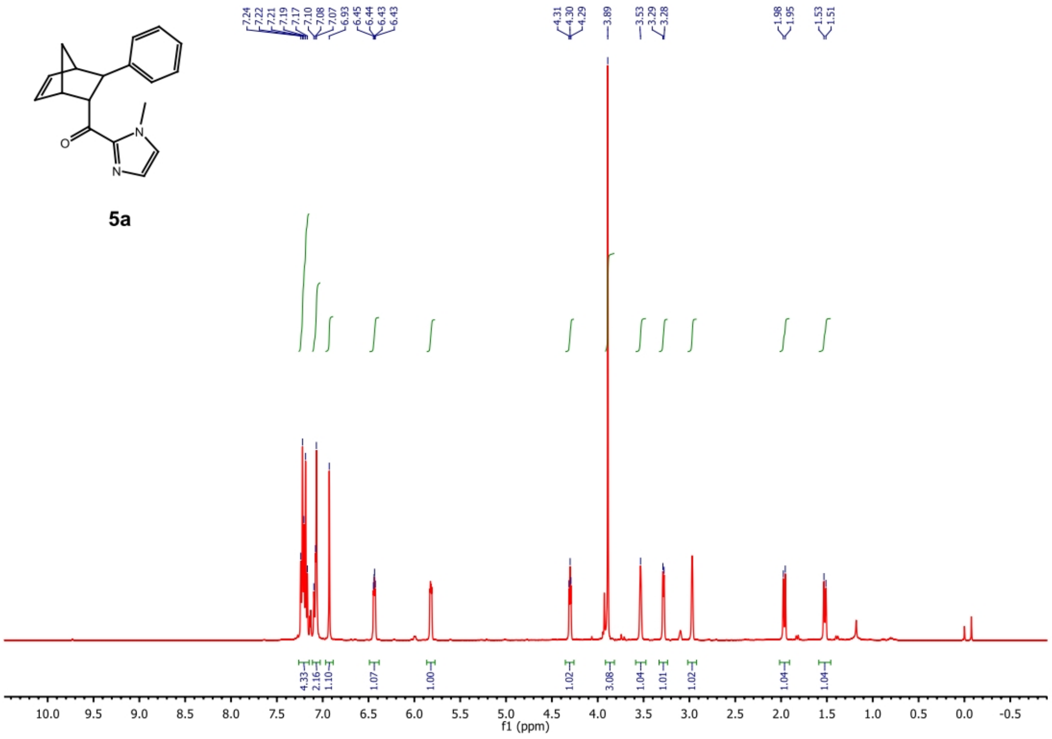

(1-methyl-1H-imidazol-2-yl)(3-p-tolylbicyclo[2.2.1]hept-5-en-2-yl)methanone (**5b**)

^{1}H NMR (400 MHz, $CDCl_3$) *endo* isomer: δ = 7.21 (d, J=7.8, 2H), 7.17 – 7.05 (m, 2H), 7.05 – 6.97 (m, 2H), 6.55 – 6.45 (m, 1H), 5.89 (dd, J=5.3, 2.4, 1H), 4.42 – 4.34 (m, 1H), 3.95 (s, 3H), 3.60 (s, 1H), 3.32 (d, J=5.0, 1H), 3.00 (s, 1H), 2.29 (s, 3H), 2.04 (d, J=8.4, 1H), 1.58 (d, J=8.5, 1H). *exo isomer*: δ = 7.17 – 7.05 (m, 5H), 7.05 – 6.97 (m, 1H), 6.55 – 6.45 (m, 1H), 6.11 – 6.04 (m, 1H), 4.42 – 4.34 (m, 1H), 3.99 (m, 4H), 3.16 (m, 2H), 2.27 (s, 3H), 2.04 (d, J=8.4, 1H), 1.46 (d, J=8.5, 1H).

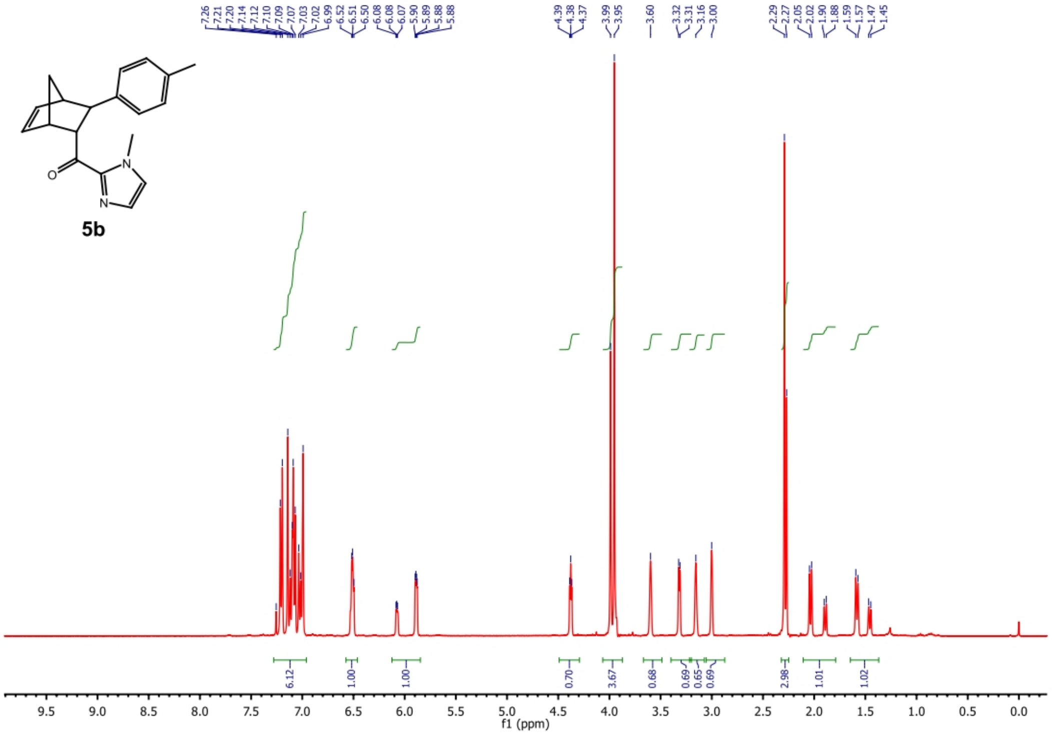

(3-(4-methoxyphenyl)bicyclo[2.2.1]hept-5-en-2-yl)(1-methyl-1H-imidazol-2-yl)methanone (**5c**)

^{1}H NMR (400 MHz, $CDCl_3$, *endo* isomer): δ 7.15 (d, *J* = 8.6 Hz, 2H), 7.07 (s, 1H), 6.92 (s, 1H), 6.74 (d, *J* = 8.7 Hz, 2H), 6.42 (dd, *J* = 5.5, 3.2 Hz, 1H), 5.81 (dd, *J* = 5.6, 2.7 Hz, 1H), 4.27 (dd, *J* = 5.2, 3.5 Hz, 1H), 3.89 (s, 3H), 3.69 (s, 3H), 3.51 (s, 1H), 3.22 (d, *J* = 4.8 Hz, 1H), 2.91 (s, 1H), 1.95 (d, *J* = 8.5 Hz, 1H), 1.51 (dd, *J* = 8.6, 1.5 Hz, 1H).

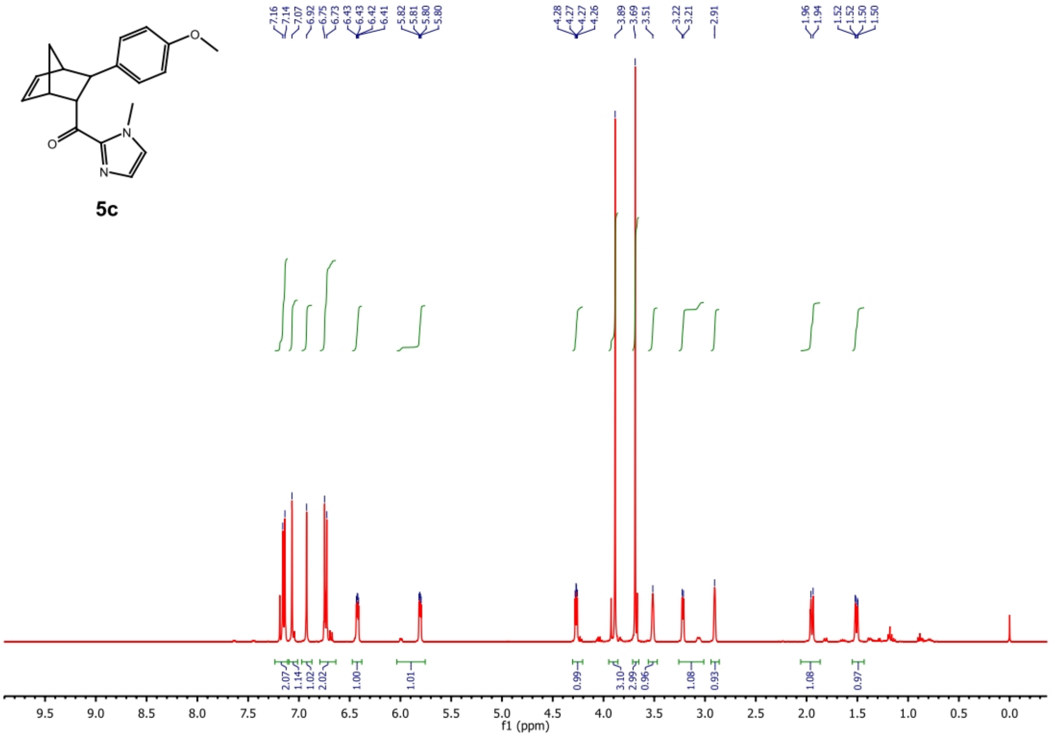

(3-(4-chlorophenyl)bicyclo[2.2.1]hept-5-en-2-yl)(1-methyl-1H-imidazol-2-yl)methanone (**5d**)

^{1}H NMR (400 MHz, $CDCl_3$, *endo* isomer): δ 7.20 – 7.05 (m, 5H), 6.94 (s, 1H), 6.42 (dd, *J* = 5.6, 3.2 Hz, 1H), 5.82 (dd, *J* = 5.6, 2.8 Hz, 1H), 4.23 (dd, *J* = 5.2, 3.5 Hz, 1H), 3.88 (d, *J* = 7.8 Hz, 3H), 3.53 (s, 1H), 3.24 (dd, *J* = 5.3, 1.6 Hz, 1H), 2.93 (d, *J* = 1.3 Hz, 1H), 1.90 (d, *J* = 8.6 Hz, 1H), 1.53 (dd, *J* = 8.6, 1.6 Hz, 1H).

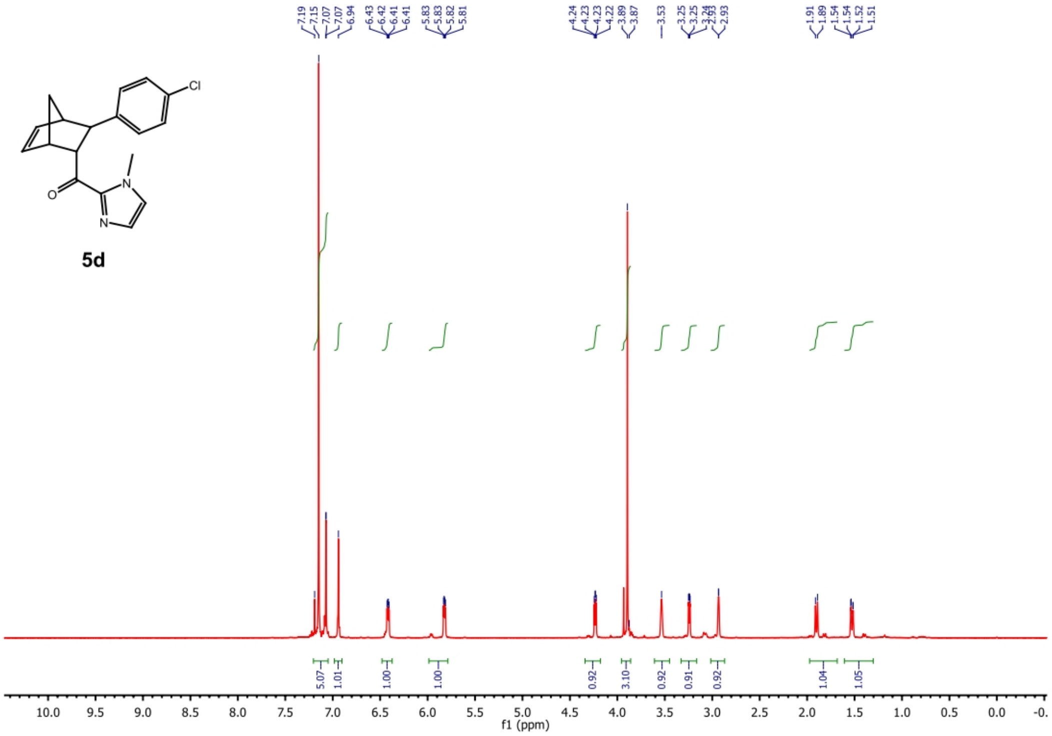

(3-(4-bromophenyl)bicyclo[2.2.1]hept-5-en-2-yl)(1-methyl-1H-imidazol-2-yl)methanone (**5e**)

^{1}H NMR (400 MHz, $CDCl_3$, *endo* isomer): δ 7.43 – 7.29 (m, 2H), 7.21 – 7.05 (m, 3H), 7.01 (s, 1H), 6.58 – 6.39 (m, 1H), 5.90 (dd, *J* = 5.3, 2.5 Hz, 1H), 4.42 – 4.25 (m, 1H), 3.96 (s, 3H), 3.61 (s, 1H), 3.30 (d, *J* = 5.1 Hz, 1H), 3.00 (s, 1H), 1.97 (d, *J* = 8.6 Hz, 1H), 1.60 (d, *J* = 8.5 Hz, 1H).

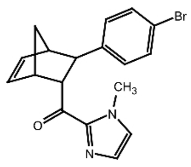

5e

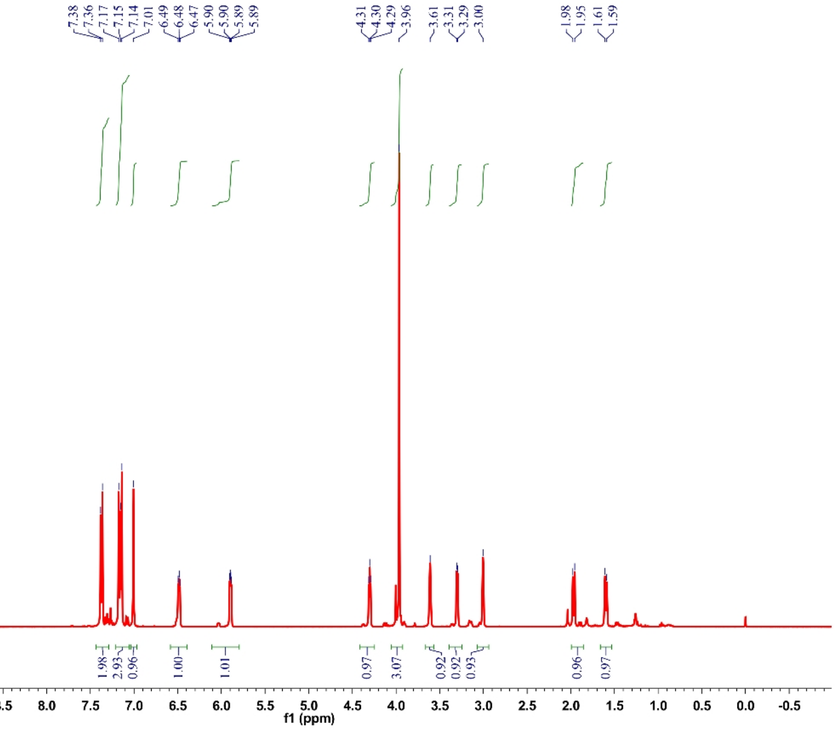

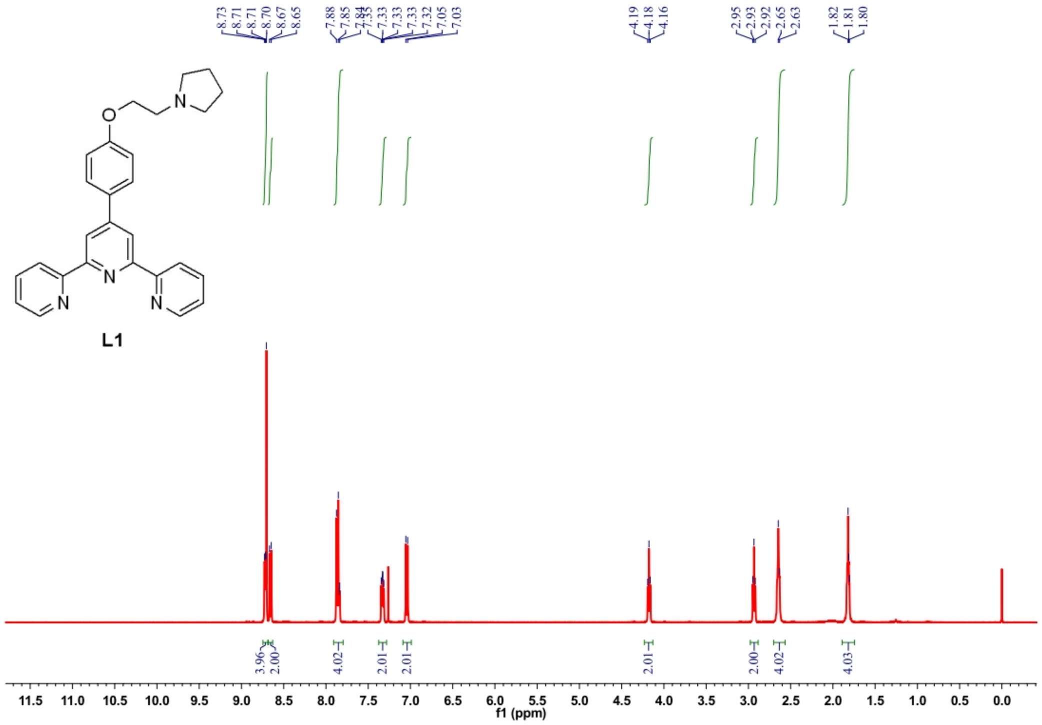
8.73
8.71
8.71
8.70
8.67
8.65
7.88
7.85
7.33
7.33
7.33
7.32
7.05
7.03
4.19
4.18
4.16
2.95
2.93
2.92
2.65
2.63
1.82
1.81
1.80
L1
3.96
2.00
4.02
2.01
2.01
2.01
2.00
4.02
4.03
f1 (ppm)

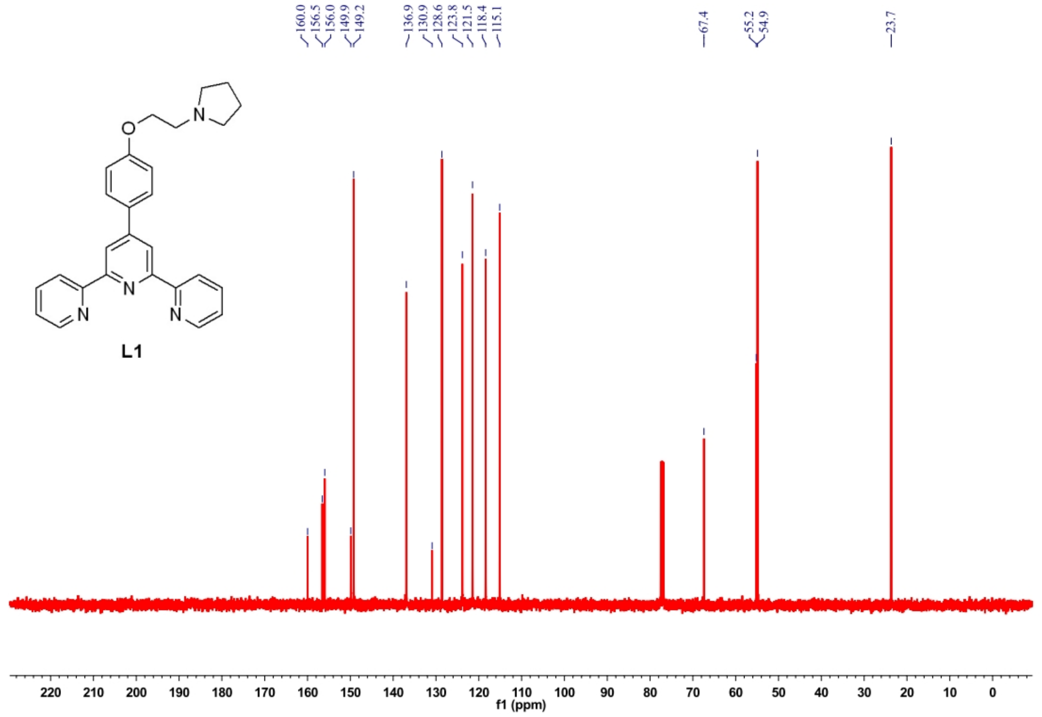
160.0
156.5
156.0
149.9
149.2
136.9
130.9
128.6
123.8
121.5
118.4
115.1
67.4
55.2
54.9
23.7
L1
f1 (ppm)

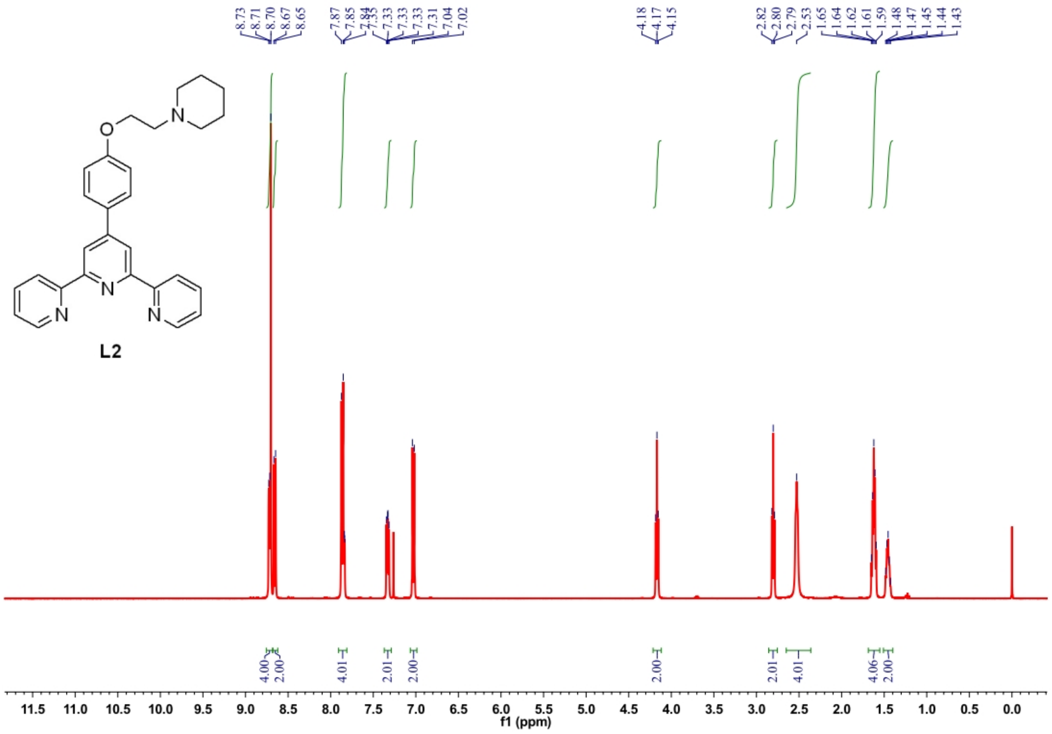

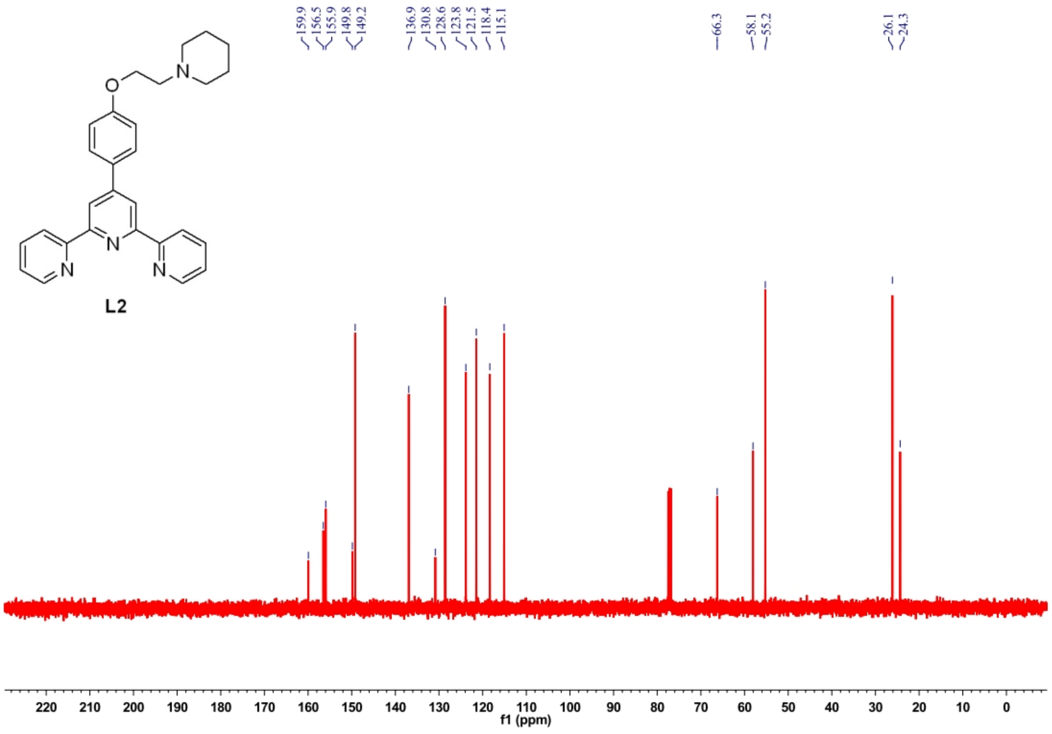

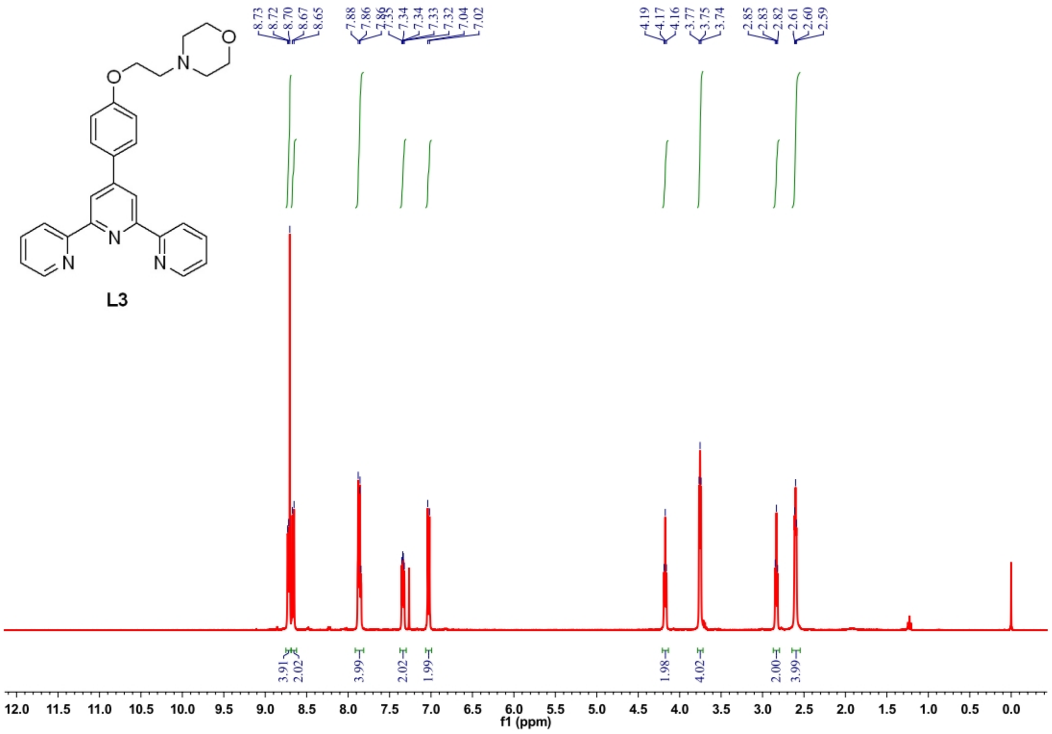
L3
8.73
8.72
8.70
8.67
8.65
7.88
7.86
7.86
7.35
7.34
7.34
7.33
7.32
7.04
7.02
4.19
4.17
4.16
3.77
3.75
3.74
2.85
2.83
2.82
2.61
2.60
2.59
3.91
2.02
3.99
2.02
1.99
1.98
4.02
2.00
3.99
f1 (ppm)

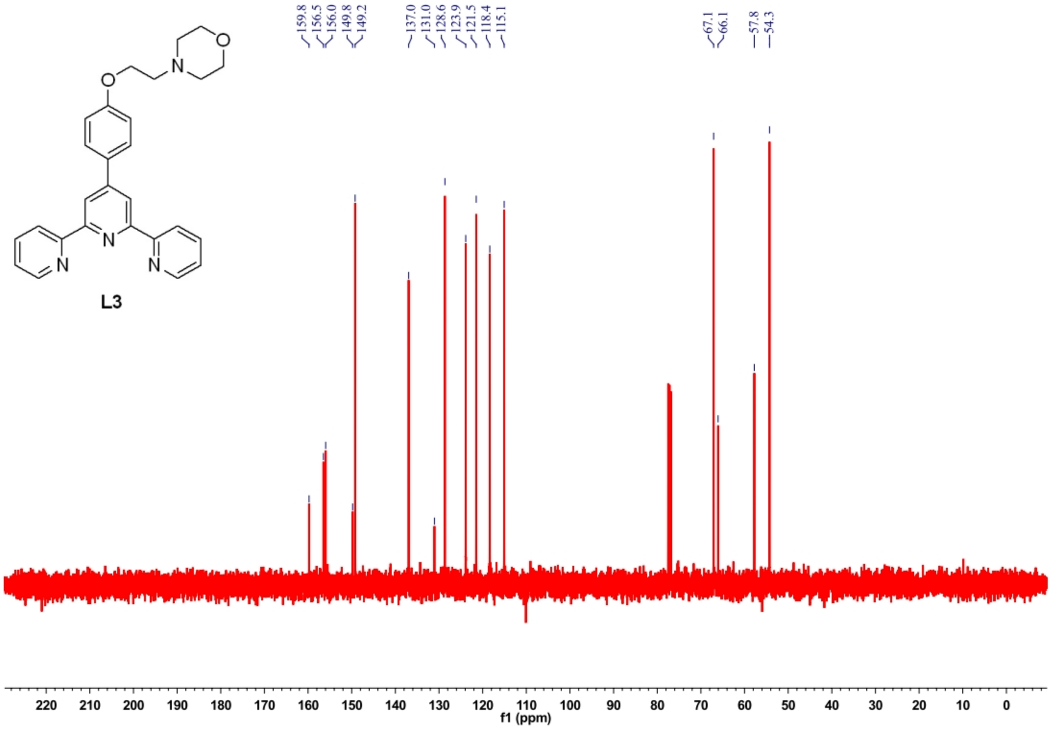
L3
159.8
156.5
156.0
149.8
149.2
137.0
131.0
128.6
123.9
121.5
118.4
115.1
67.1
66.1
57.8
54.3
f1 (ppm)

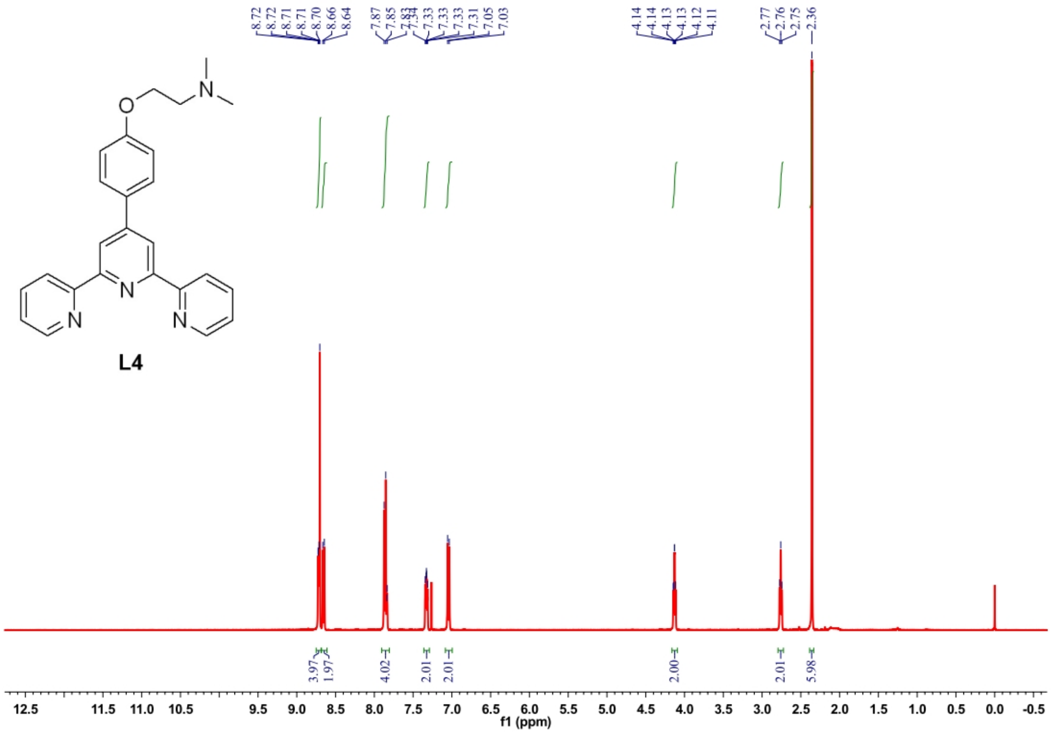
L4
8.72
8.72
8.71
8.71
8.70
8.66
8.64
7.87
7.85
7.33
7.33
7.33
7.31
7.05
7.03
4.14
4.14
4.13
4.13
4.12
4.11
2.77
2.76
2.75
2.36
3.97
1.97
4.02
2.01
2.01
2.00
2.01
5.98
f1 (ppm)

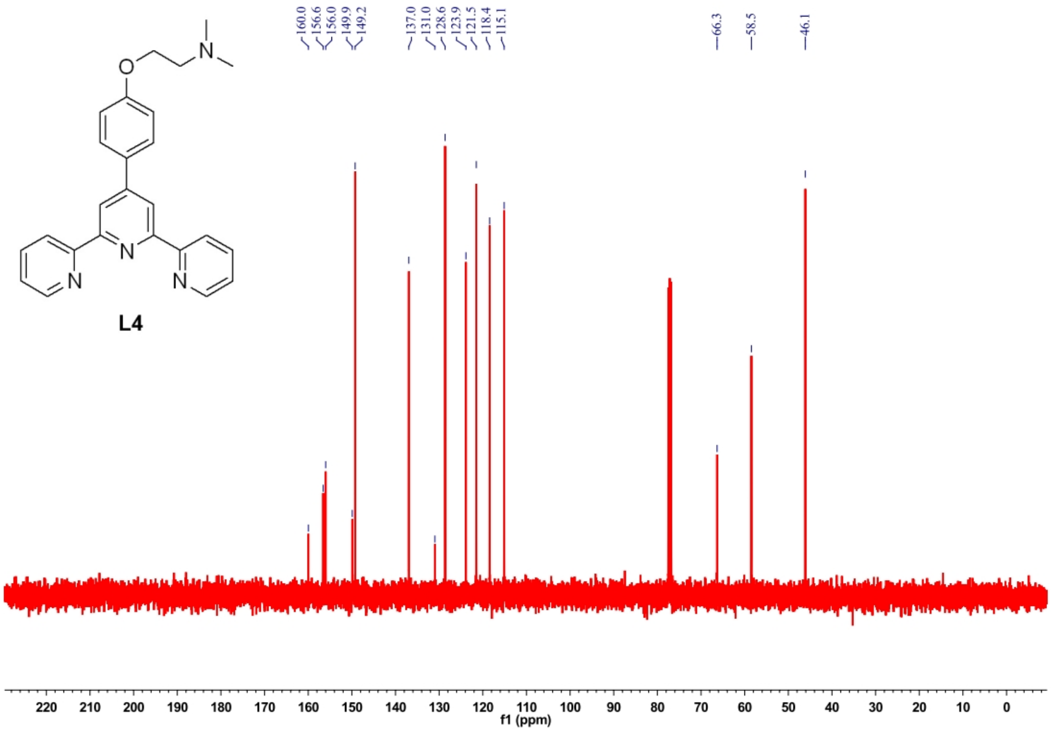
L4
160.0
156.6
156.0
149.9
149.2
137.0
131.0
128.6
123.9
121.5
118.4
115.1
66.3
58.5
46.1
f1 (ppm)

HPLC Traces of Products

1. Product **3a**

(1) Racemic **3a**

Retention times: 13.0, 14.7 (*exo* isomer) and 17.0, 22.0 (*endo* isomer) mins

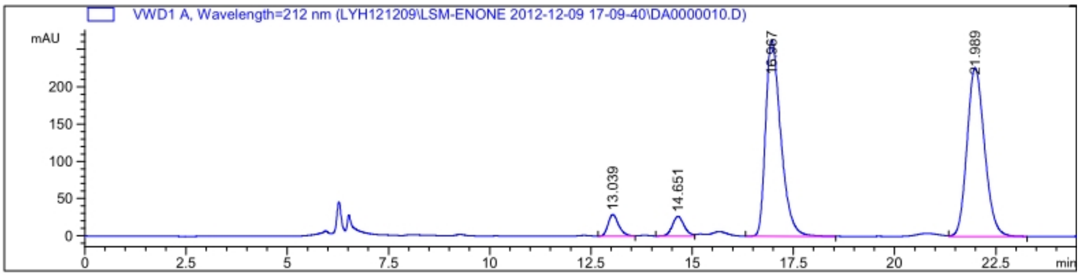

===

Area Percent Report

===

Sorted By : Signal
Multiplier: : 1.0000
Dilution: : 1.0000
Use Multiplier & Dilution Factor with ISTDs

Signal 1: VWD1 A, Wavelength=212 nm

Peak #	RetTime [min]	Type	Width [min]	Area mAU *s	Height [mAU]	Area %
1	13.039	VV	0.2821	545.54974	29.38276	3.6834
2	14.651	VV	0.3169	557.33136	26.79624	3.7630
3	16.967	VB	0.4002	6895.75879	263.32379	46.5584
4	21.989	VB	0.4662	6812.34863	226.33138	45.9952

Totals : 1.48110e4 545.83416

===

*** End of Report ***

(2) Product **3a** from the Diels-Alder reaction catalyzed by HT21-NH4-CuL1 (*endo* isomer: 94% *ee*)

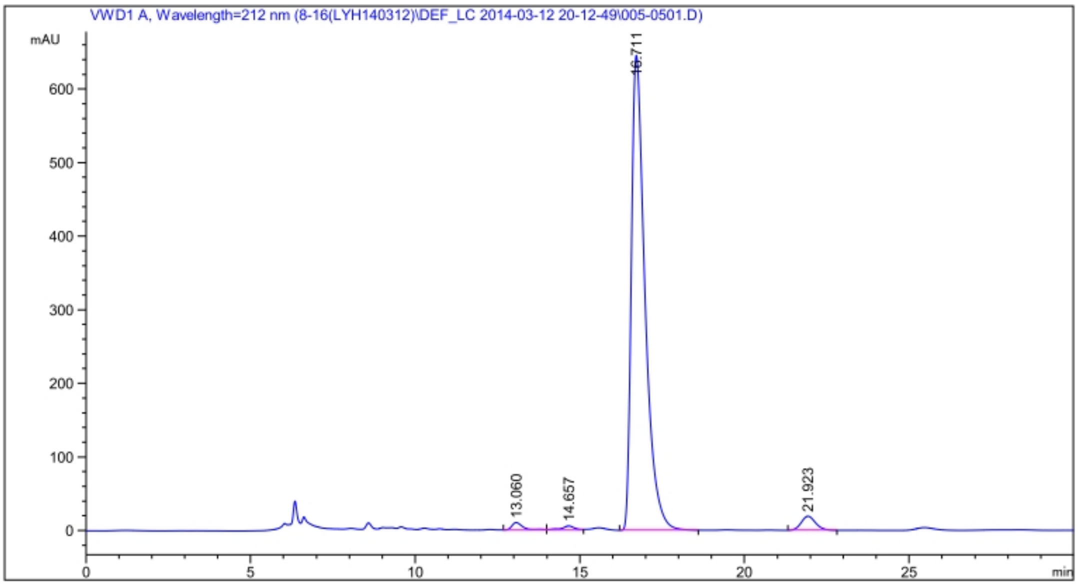

==

面积百分比报告

==

排序 : 信号
乘积因子 : 1.0000
稀释因子 : 1.0000
内标使用乘积因子和稀释因子

信号 1: VWD1 A, Wavelength=212 nm

峰 #	保留时间 [min]	类型	峰宽 [min]	峰面积 mAU *s	峰高 [mAU]	峰面积 %
1	13.060	VV	0.3693	257.80942	10.34860	1.3184
2	14.657	VV	0.4261	175.22066	5.80997	0.8961
3	16.711	VB	0.4314	1.85428e4	645.19928	94.8286
4	21.923	BB	0.4650	578.18036	19.11723	2.9568

(3) Product **3a** from the Diels-Alder reaction catalyzed by L-HT21-NH4-CuL1 (*endo* isomer: -92% *ee*)

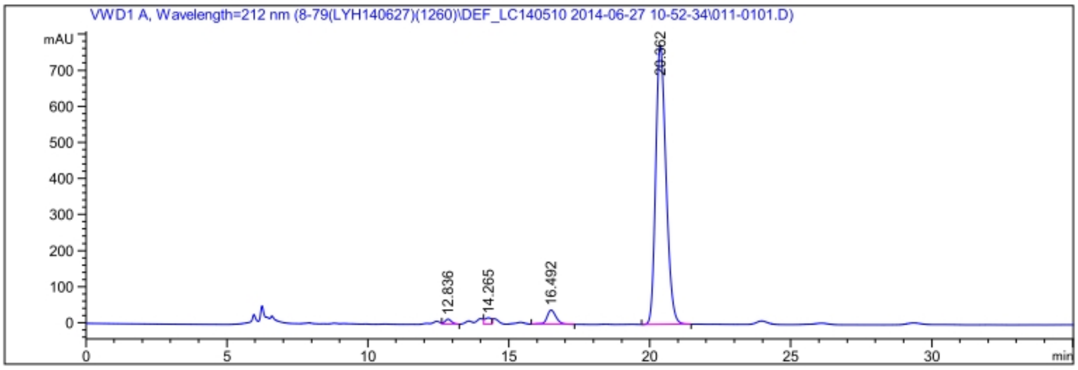

==

面积百分比报告

==

排序 : 信号
乘积因子 : 1.0000
稀释因子 : 1.0000
内标使用乘积因子和稀释因子

信号 1: VWD1 A, Wavelength=212 nm

峰 #	保留时间 [min]	类型	峰宽 [min]	峰面积 [mAU*s]	峰高 [mAU]	峰面积 %
1	12.836	VV	0.2516	244.88458	14.56330	1.1630
2	14.265	VV	0.2383	305.47574	18.18080	1.4508
3	16.492	VB	0.3457	902.52008	39.65071	4.2864
4	20.362	VB	0.3917	1.96027e4	773.94250	93.0998

总量 : 2.10556e4 846.33732

==

2. Product **3b**

(1) Racemic **3b**

Retention times: 11.0, 12.6 (*exo* isomer) and 13.4, 18.1 (*endo* isomer) mins

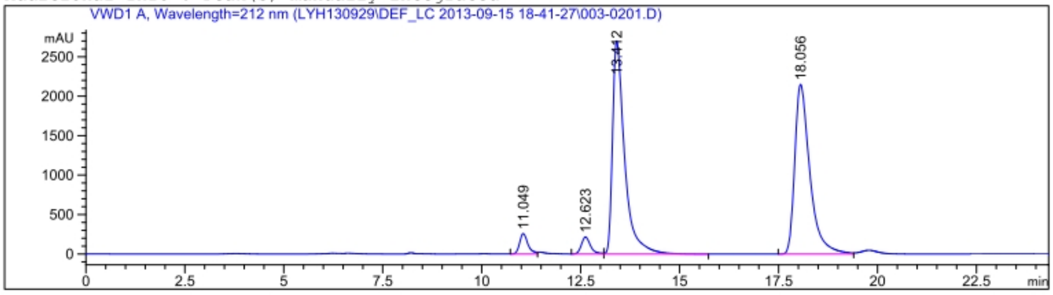


```
=====================================================================
                          Area Percent Report
=====================================================================

Sorted By             :      Signal
Multiplier:                   :      1.0000
Dilution:                     :      1.0000
Use Multiplier & Dilution Factor with ISTDs


Signal 1: VWD1 A, Wavelength=212 nm
```

Peak #	RetTime [min]	Type	Width [min]	Area [mAU*s]	Height [mAU]	Area %
1	11.049	VV	0.2193	3786.92505	260.22565	3.1164
2	12.623	VV	0.2440	3466.84985	216.15584	2.8530
3	13.412	VB	0.3139	5.71527e4	2701.50098	47.0337
4	18.056	VV	0.3987	5.71079e4	2151.03516	46.9969
Totals :				1.21514e5	5328.91762	

```
=====================================================================
                          *** End of Report ***
```

(2) Product **3b** from the Diels-Alder reaction catalyzed by HT21-NH4-CuL1 (*endo* isomer: 96% *ee*)

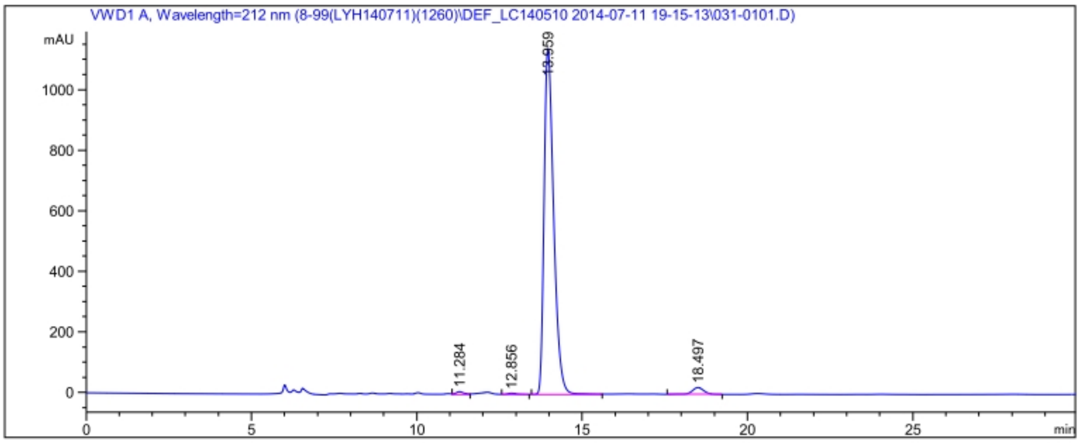

面积百分比报告

排序 : 信号
乘积因子 : 1.0000
稀释因子 : 1.0000
内标使用乘积因子和稀释因子

信号 1: VWD1 A, Wavelength=212 nm

峰 #	保留时间 [min]	类型	峰宽 [min]	峰面积 [mAU*s]	峰高 [mAU]	峰面积 %
1	11.284	VV	0.2662	183.02797	9.92121	0.7637
2	12.856	VB	0.3407	114.43951	4.75194	0.4775
3	13.959	BB	0.3087	2.30806e4	1141.76819	96.3063
4	18.497	VB	0.3937	587.75977	22.82935	2.4525

总量 : 2.39658e4 1179.27069

3. Product **3c**

(1) Racemic **3c**

Retention times: 13.8, 14.2 (*exo* isomer) and 16.6, 22.8 (*endo* isomer) mins

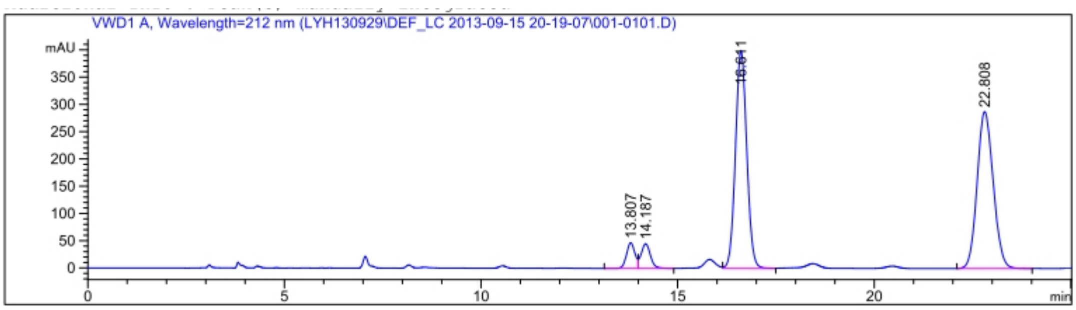


```
=====================================================================
                         Area Percent Report
=====================================================================

Sorted By              :      Signal
Multiplier:                   :      1.0000
Dilution:                     :      1.0000
Use Multiplier & Dilution Factor with ISTDs


Signal 1: VWD1 A, Wavelength=212 nm

Peak RetTime Type  Width     Area      Height     Area
  #   [min]        [min]   [mAU*s]     [mAU]       %
----|-------|----|-------|----------|----------|--------|
   1  13.807 BV    0.2309  708.48175   47.10786   4.0206
   2  14.187 VB    0.2528  748.52386   45.24762   4.2478
   3  16.611 VB    0.3120 7994.28027  399.87192  45.3670
   4  22.808 BB    0.4420 8170.07568  287.82578  46.3646

Totals :                  1.76214e4   780.05318


=====================================================================
                          *** End of Report ***
```

(2) Product **3c** from the Diels-Alder reaction catalyzed by HT21-NH4-CuL1 (*endo* isomer: 98% *ee*)

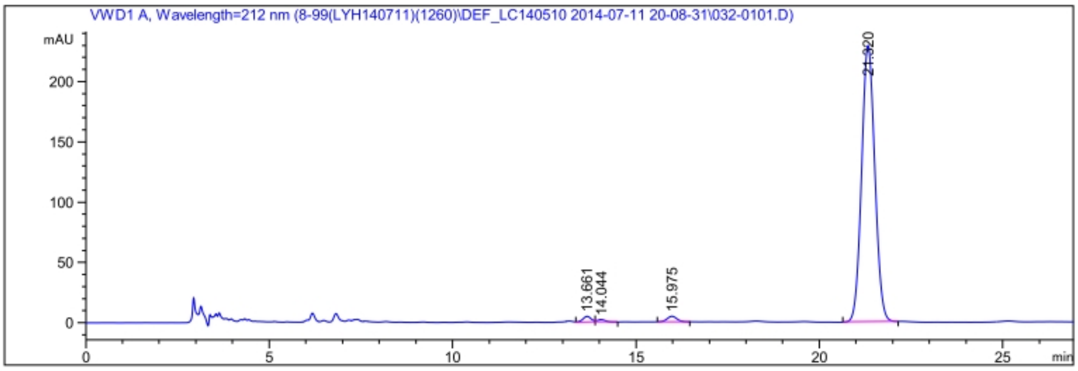

===

面积百分比报告

===

排序 : 信号
乘积因子 : 1.0000
稀释因子 : 1.0000
内标使用乘积因子和稀释因子

信号 1: VWD1 A, Wavelength=212 nm

峰 #	保留时间 [min]	类型	峰宽 [min]	峰面积 [mAU*s]	峰高 [mAU]	峰面积 %
1	13.661	VV	0.2450	77.32947	4.83356	1.3055
2	14.044	VB	0.2372	32.54331	2.08844	0.5494
3	15.975	BB	0.2897	86.83061	4.64072	1.4659
4	21.320	BB	0.3893	5726.47998	229.08356	96.6791

总量 : 5923.18337 240.64628

===

4. Product **3d**

(1) Racemic **3d**

Retention times: 20.8, 26.9 (*exo* isomer) and 25.4, 44.4 (*endo* isomer) mins

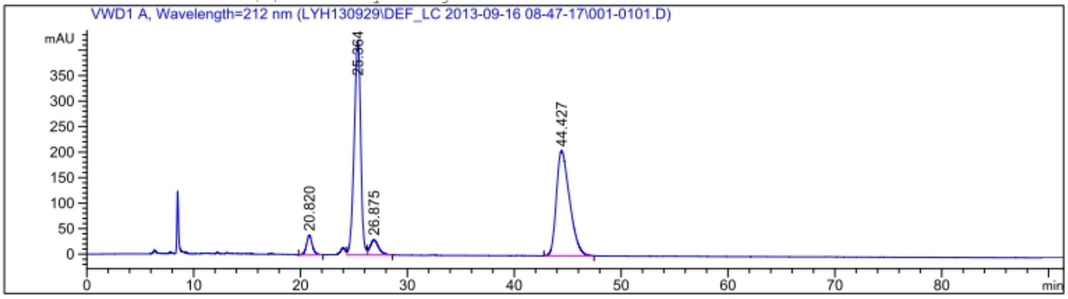

Area Percent Report

Sorted By : Signal
Multiplier: : 1.0000
Dilution: : 1.0000
Use Multiplier & Dilution Factor with ISTDs

Signal 1: VWD1 A, Wavelength=212 nm

Peak #	RetTime [min]	Type	Width [min]	Area [mAU*s]	Height [mAU]	Area %
1	20.820	BB	0.5783	1459.81567	38.59794	3.7885
2	25.364	VV	0.6586	1.77287e4	418.76682	46.0090
3	26.875	VB	0.8094	1611.82507	29.70839	4.1830
4	44.427	BB	1.3103	1.77327e4	206.02675	46.0196
Totals :				3.85330e4	693.09989	

*** End of Report ***

(2) Product **3d** from the Diels-Alder reaction catalyzed by HT21-NH4-CuL1 (*endo* isomer: 93% *ee*)

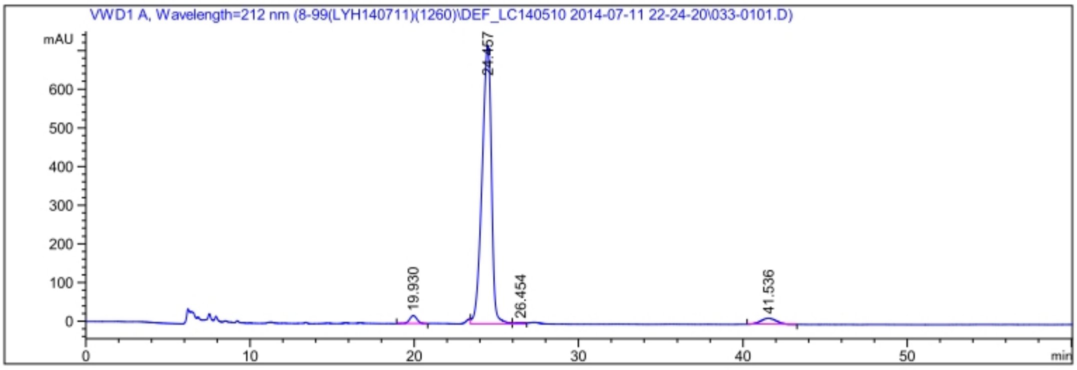

===

面积百分比报告

===

排序 : 信号
乘积因子 : 1.0000
稀释因子 : 1.0000
内标使用乘积因子和稀释因子

信号 1: VWD1 A, Wavelength=212 nm

峰 #	保留时间 [min]	类型	峰宽 [min]	峰面积 [mAU*s]	峰高 [mAU]	峰面积 %
1	19.930	BB	0.4927	654.14752	20.77306	2.1491
2	24.457	VB	0.6143	2.85767e4	720.53961	93.8845
3	26.454	BV	0.6010	162.57109	3.53301	0.5341
4	41.536	BB	0.9626	1044.74170	15.76636	3.4323

总量 : 3.04382e4 760.61205

===

5. Product **3e**

(1) Racemic **3e**

Retention times: 12.5, 16.0 (*exo* isomer) and 14.6, 18.1 (*endo* isomer) mins

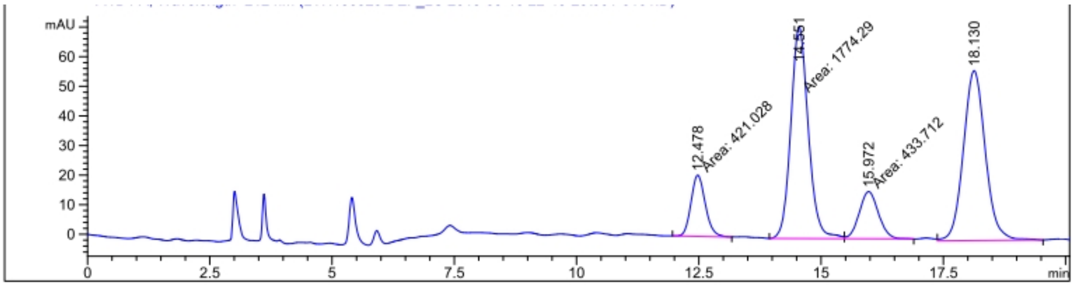

Area Percent Report

Sorted By : Signal
Multiplier: : 1.0000
Dilution: : 1.0000
Use Multiplier & Dilution Factor with ISTDs

Signal 1: VWD1 A, Wavelength=212 nm

Peak #	RetTime [min]	Type	Width [min]	Area [mAU*s]	Height [mAU]	Area %
1	12.478	MM	0.3388	421.02759	20.71424	9.5208
2	14.551	MF	0.4143	1774.29187	71.37769	40.1225
3	15.972	FM	0.4531	433.71246	15.95338	9.8077
4	18.130	VV	0.4826	1793.14966	57.39640	40.5490

Totals : 4422.18158 165.44171

*** End of Report ***

(2) Product **3e** from the Diels-Alder reaction catalyzed by HT21-NH4-CuL1 (*endo* isomer: 99% *ee*)

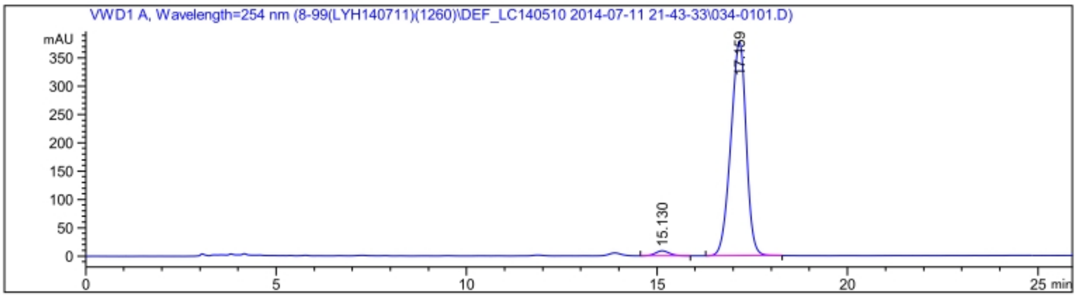

面积百分比报告

排序 : 信号
乘积因子 : 1.0000
稀释因子 : 1.0000
内标使用乘积因子和稀释因子

信号 1: VWD1 A, Wavelength=254 nm

峰 #	保留时间 [min]	类型	峰宽 [min]	峰面积 [mAU*s]	峰高 [mAU]	峰面积 %
1	15.130	BB	0.3914	223.85844	8.76132	2.0660
2	17.159	BB	0.4373	1.06117e4	377.44269	97.9340

总量 : 1.08355e4 386.20401

6. Product **3f**

(1) Racemic **3f**

Retention times: 7.4 and 10.8 mins

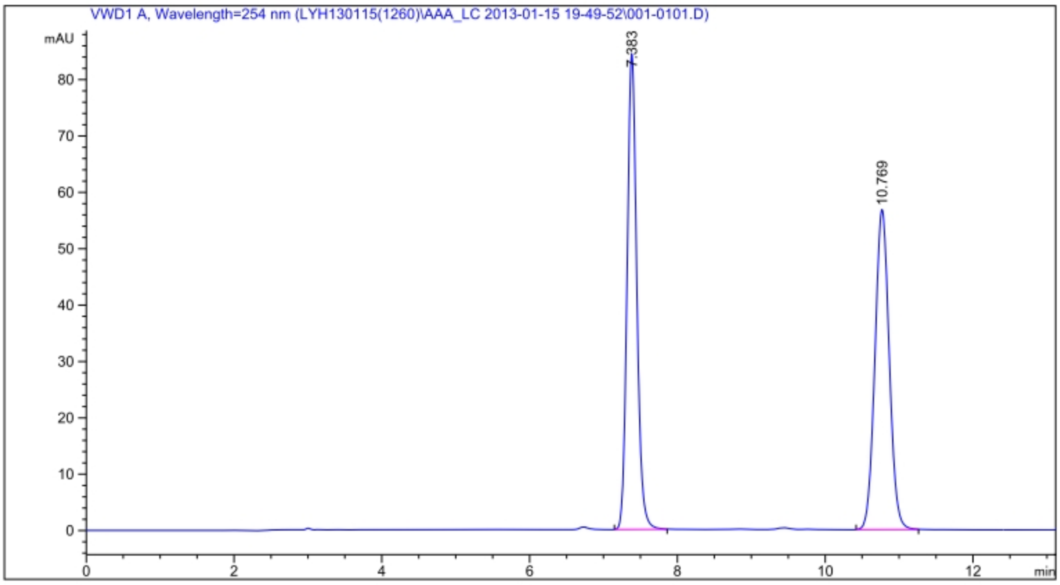


```
=====================================================================
                         Area Percent Report
=====================================================================

Sorted By             :      Signal
Multiplier:                   :      1.0000
Dilution:                     :      1.0000
Use Multiplier & Dilution Factor with ISTDs


Signal 1: VWD1 A, Wavelength=254 nm

Peak RetTime Type  Width     Area      Height     Area
  #   [min]        [min]   [mAU*s]     [mAU]       %
----|-------|----|-------|----------|----------|--------|
   1   7.383 BB    0.1393  764.62390   84.33156  49.9907
   2  10.769 BB    0.2097  764.90961   56.76023  50.0093

Totals :                  1529.53351  141.09179

=====================================================================
                          *** End of Report ***
```

(2) Product **3f** from the Diels-Alder reaction catalyzed by HT21-NH4-CuL1 (*endo* isomer: 98% *ee*)

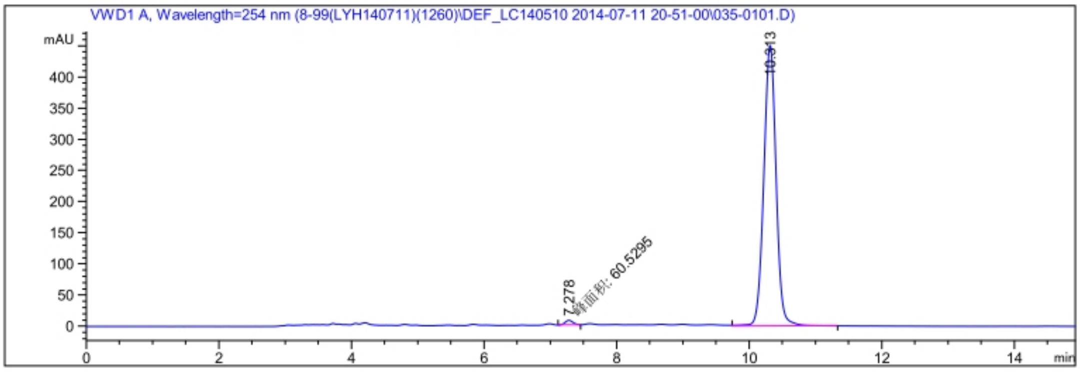

===

面积百分比报告

===

排序 : 信号
乘积因子 : 1.0000
稀释因子 : 1.0000
内标使用乘积因子和稀释因子

信号 1: VWD1 A, Wavelength=254 nm

峰 #	保留时间 [min]	类型	峰宽 [min]	峰面积 [mAU*s]	峰高 [mAU]	峰面积 %
1	7.278	MM	0.1379	60.52946	7.31544	1.0320
2	10.313	VB	0.1985	5804.98926	450.19852	98.9680
总量 :				5865.51872	457.51395	

===

7. Product **5a**

(1) Racemic **5a**

Retention times: 21.9, 27.1 (*exo* isomer) and 25.1, 35.1 (*endo* isomer) mins

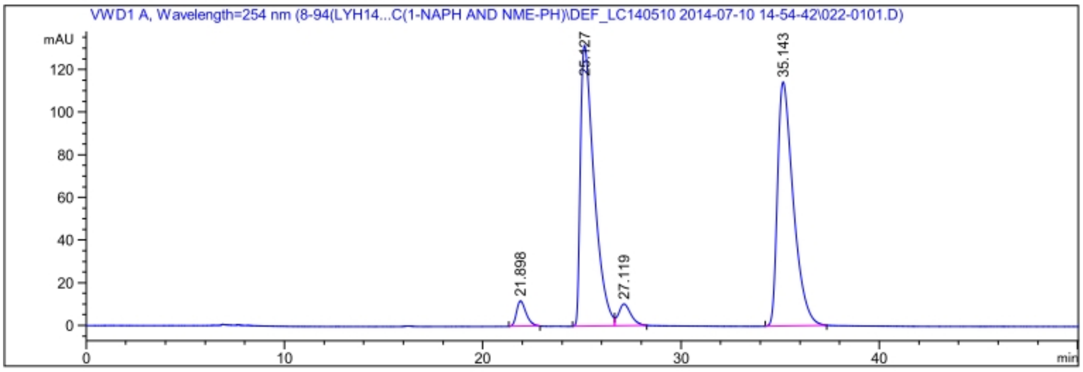

面积百分比报告

排序 : 信号
乘积因子 : 1.0000
稀释因子 : 1.0000
内标使用乘积因子和稀释因子

信号 1: VWD1 A, Wavelength=254 nm

峰 #	保留时间 [min]	类型	峰宽 [min]	峰面积 [mAU*s]	峰高 [mAU]	峰面积 %
1	21.898	BB	0.5063	403.26956	11.93801	2.9921
2	25.127	BV	0.7178	6307.14355	131.65181	46.7968
3	27.119	VB	0.6349	439.48395	10.23832	3.2608
4	35.143	BB	0.8303	6327.81787	114.42680	46.9502

总量 : 1.34777e4 268.25494

(2) Product **5a** from the Diels-Alder reaction catalyzed by HT21-NH4-CuL1 (*endo* isomer: 98% *ee*)

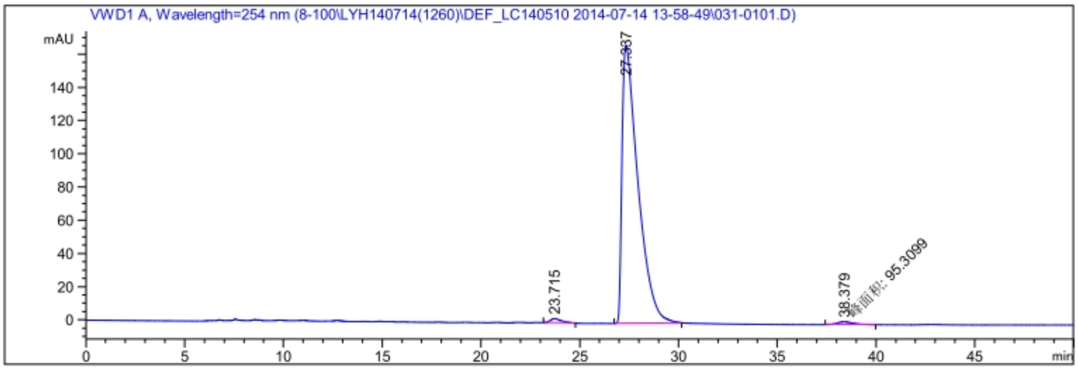

==

面积百分比报告

==

排序 : 信号
乘积因子 : 1.0000
稀释因子 : 1.0000
内标使用乘积因子和稀释因子

信号 1: VWD1 A, Wavelength=254 nm

峰 #	保留时间 [min]	类型	峰宽 [min]	峰面积 [mAU*s]	峰高 [mAU]	峰面积 %
1	23.715	BB	0.5441	101.82387	2.52898	1.0641
2	27.337	BB	0.8175	9371.50879	167.48953	97.9398
3	38.379	MM	0.9306	95.30985	1.70703	0.9961

总量 : 9568.64251 171.72554

==

8. Product **5b**

(1) Racemic **5b**

Retention times: 30.0, 35.5 (*exo* isomer) and 37.5, 40.3 (*endo* isomer) mins

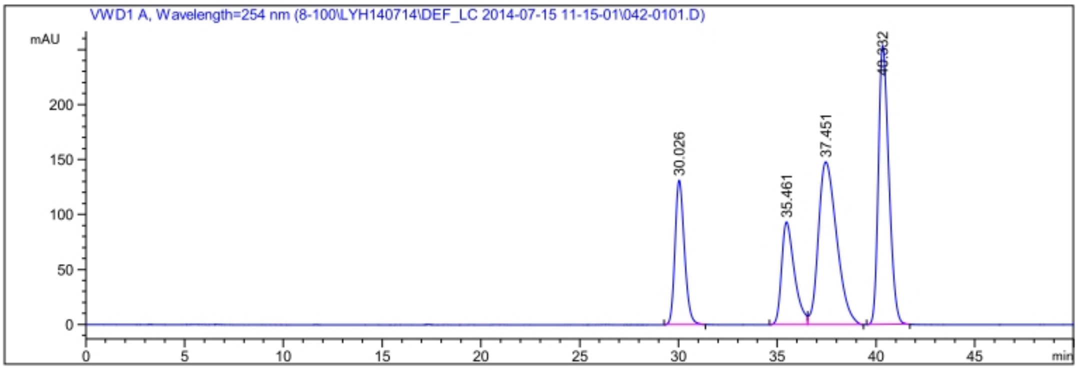

面积百分比报告

排序 : 信号
乘积因子 : 1.0000
稀释因子 : 1.0000
内标使用乘积因子和稀释因子

信号 1: VWD1 A, Wavelength=254 nm

峰 #	保留时间 [min]	类型	峰宽 [min]	峰面积 mAU *s	峰高 [mAU]	峰面积 %
1	30.026	BB	0.5077	4340.55664	131.41245	15.8289
2	35.461	BV	0.6773	4233.79053	93.40224	15.4396
3	37.451	VB	0.9911	9511.67090	148.08525	34.6867
4	40.332	BB	0.5671	9335.68457	254.02907	34.0449

总量 : 2.74217e4 626.92901

(2) Product **5b** from the Diels-Alder reaction catalyzed by HT21-NH4-CuL1 (*endo* isomer: 94% *ee*)

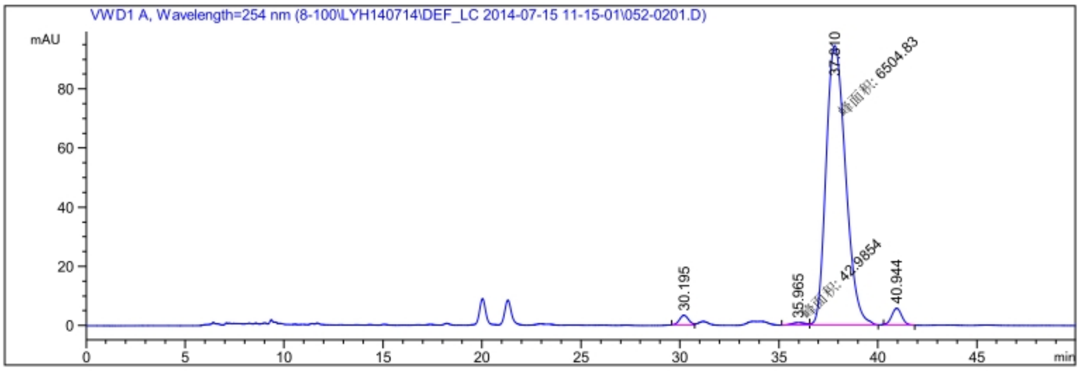

面积百分比报告

排序 : 信号
乘积因子 : 1.0000
稀释因子 : 1.0000
内标使用乘积因子和稀释因子

信号 1: VWD1 A, Wavelength=254 nm

峰 #	保留时间 [min]	类型	峰宽 [min]	峰面积 mAU *s	峰高 [mAU]	峰面积 %
1	30.195	BV	0.5047	107.16024	3.32005	1.5619
2	35.965	MF	0.8175	42.98543	8.76339e-1	0.6265
3	37.810	FM	1.1464	6504.83301	94.57201	94.8076
4	40.944	BB	0.5524	206.10716	5.76660	3.0040

总量 : 6861.08584 104.53500

9. Product **5c**

(1) Racemic **5c**

Retention times: 14.4, 21.6 (*exo* isomer) and 17.4, 28.0 (*endo* isomer) mins

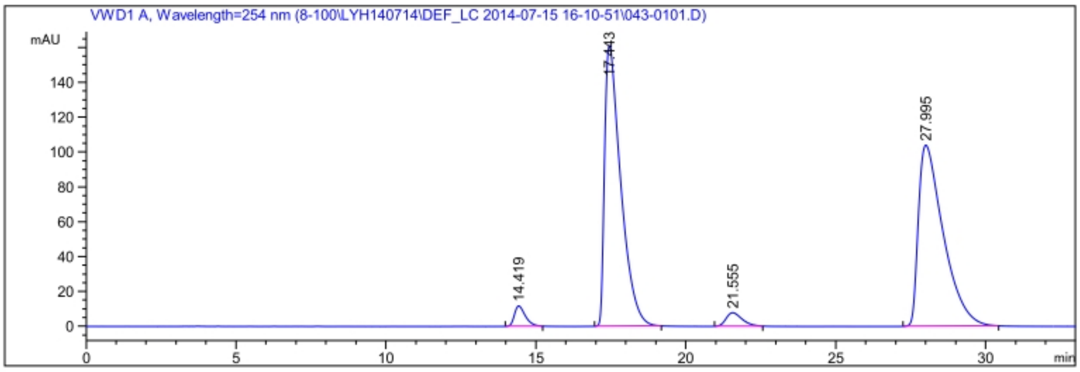

面积百分比报告

排序 : 信号
乘积因子 : 1.0000
稀释因子 : 1.0000
内标使用乘积因子和稀释因子

信号 1: VWD1 A, Wavelength=254 nm

峰 #	保留时间 [min]	类型	峰宽 [min]	峰面积 mAU *s	峰高 [mAU]	峰面积 %
1	14.419	BB	0.3859	297.72125	11.69005	2.3570
2	17.443	BB	0.5625	6034.53516	161.01726	47.7733
3	21.555	BB	0.5648	292.44934	7.81286	2.3152
4	27.995	BB	0.8605	6006.88916	103.96090	47.5545

总量 : 1.26316e4 284.48107

(2) Product **5c** from the Diels-Alder reaction catalyzed by HT21-NH4-CuL1 (*endo* isomer: >99% *ee*)

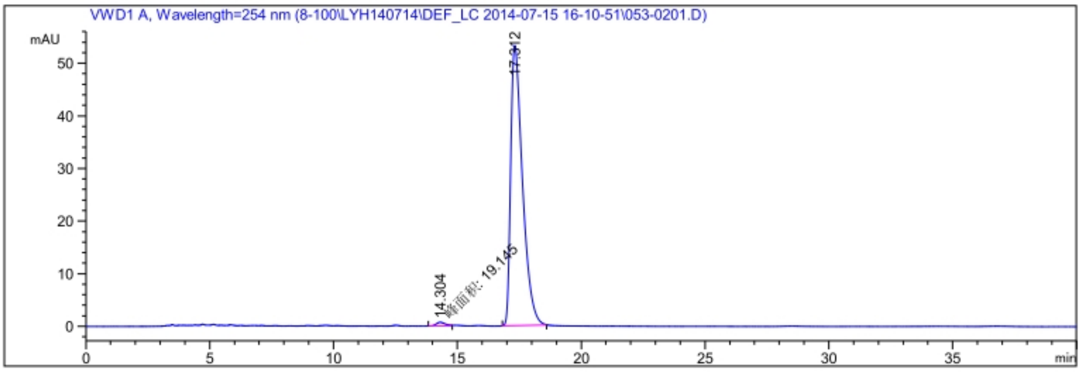

===

面积百分比报告

===

排序 : 信号
乘积因子 : 1.0000
稀释因子 : 1.0000
内标使用乘积因子和稀释因子

信号 1: VWD1 A, Wavelength=254 nm

峰 #	保留时间 [min]	类型	峰宽 [min]	峰面积 mAU *s	峰高 [mAU]	峰面积 %
1	14.304	MF	0.4429	19.14496	7.20490e-1	1.0755
2	17.312	BB	0.5007	1760.96838	53.26444	98.9245

总量 : 1780.11334 53.98493

===

10. Product **5d**

(1) Racemic **5d**

Retention times: 6.4, 9.9 (*exo* isomer) and 6.8, 8.8 (*endo* isomer) mins

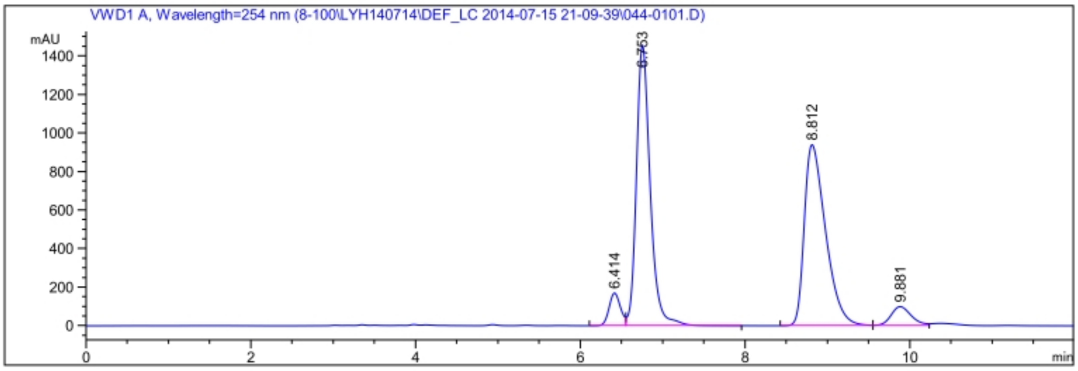

面积百分比报告

排序 : 信号
乘积因子 : 1.0000
稀释因子 : 1.0000
内标使用乘积因子和稀释因子

信号 1: VWD1 A, Wavelength=254 nm

峰 #	保留时间 [min]	类型	峰宽 [min]	峰面积 mAU *s	峰高 [mAU]	峰面积 %
1	6.414	VV	0.1485	1628.29016	169.52763	4.3853
2	6.753	VB	0.1763	1.69098e4	1455.40881	45.5416
3	8.812	BV	0.2750	1.68661e4	939.32184	45.4239
4	9.881	VV	0.2670	1726.27332	99.22031	4.6492

总量 : 3.71304e4 2663.47859

(2) Product **5d** from the Diels-Alder reaction catalyzed by HT21-NH4-CuL1 (*endo* isomer: 98% *ee*)

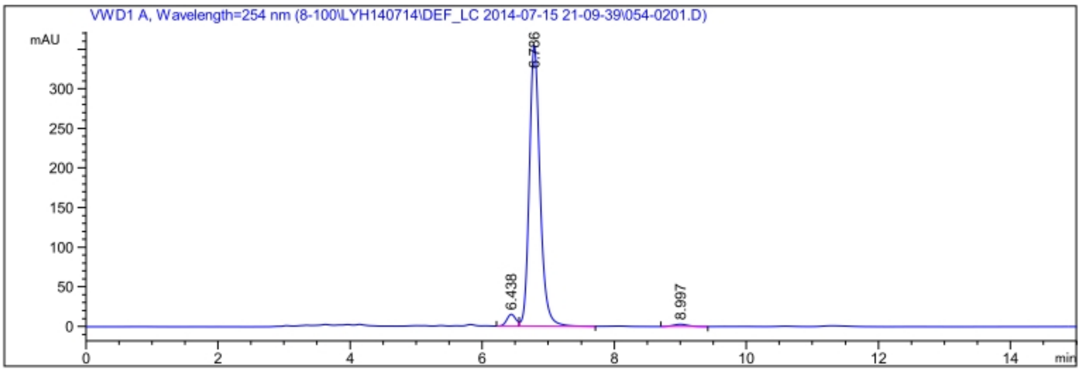

面积百分比报告

排序 : 信号
乘积因子 : 1.0000
稀释因子 : 1.0000
内标使用乘积因子和稀释因子

信号 1: VWD1 A, Wavelength=254 nm

峰 #	保留时间 [min]	类型	峰宽 [min]	峰面积 mAU *s	峰高 [mAU]	峰面积 %
1	6.438	VV	0.1497	146.81892	15.11268	3.5020
2	6.786	VB	0.1726	4001.93457	354.20807	95.4564
3	8.997	BB	0.2415	43.66702	2.80290	1.0416
总量 :				4192.42052	372.12365	

11. Product **5e**

(1) Racemic **5e**

Retention times: 15.6, 31.0 (*exo* isomer) and 17.6, 36.1 (*endo* isomer) mins

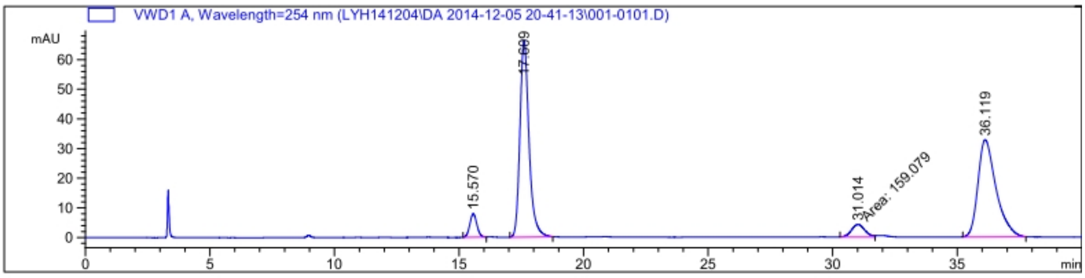

Area Percent Report

Sorted By : Signal
Multiplier: : 1.0000
Dilution: : 1.0000
Use Multiplier & Dilution Factor with ISTDs

Signal 1: VWD1 A, Wavelength=254 nm

Peak #	RetTime [min]	Type	Width [min]	Area mAU *s	Height [mAU]	Area %
1	15.570	BB	0.3057	155.33134	7.88053	4.2542
2	17.609	BB	0.3856	1678.83728	66.31950	45.9800
3	31.014	MF	0.6245	159.07932	4.24561	4.3569
4	36.119	BB	0.7537	1657.98242	32.83168	45.4089
Totals :				3651.23036	111.27731	

*** End of Report ***

(2) Product **5e** from the Diels-Alder reaction catalyzed by HT21-NH4-CuL1 (*endo* isomer: >99% *ee*)

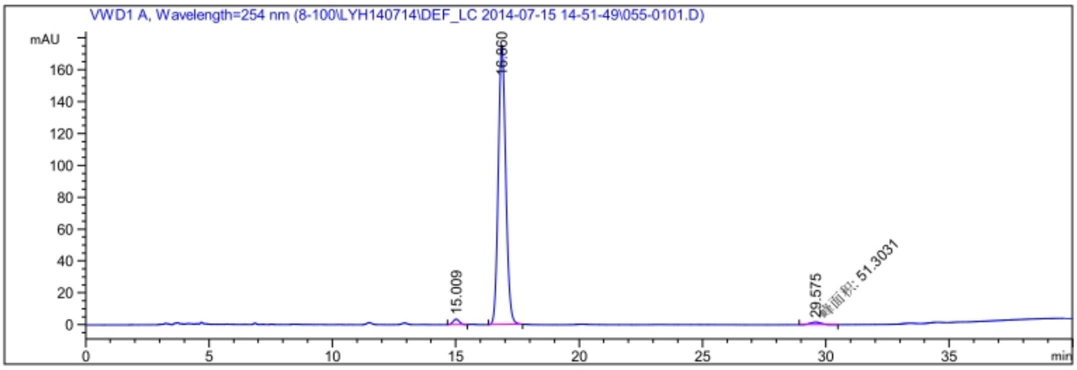

===
面积百分比报告
===

排序 : 信号
乘积因子 : 1.0000
稀释因子 : 1.0000
内标使用乘积因子和稀释因子

信号 1: VWD1 A, Wavelength=254 nm

峰 #	保留时间 [min]	类型	峰宽 [min]	峰面积 mAU *s	峰高 [mAU]	峰面积 %
1	15.009	BB	0.2716	60.61797	3.48141	1.5925
2	16.860	BB	0.3261	3694.54150	175.17296	97.0597
3	29.575	MM	0.5328	51.30312	1.60483	1.3478

总量 : 3806.46259 180.25921

===

References

1 S. Otto, F. Bertoncin and J. B. F. N. Engberts, *J. Am. Chem. Soc.*, 1996, **118**, 7702.

2 D. A. Evans, K. R. Fandrick and H.-J. Song, *J. Am. Chem. Soc.*, 2005, **127**, 8942.

3 M. C. Myers, A. R. Bharadwaj, B. C. Milgram and K. A. Scheidt, *J. Am. Chem. Soc.*, 2005, **127**, 14675.

4 G. Roelfes, A. J. Boersma and B. L. Feringa, *Chem. Commun.*, 2006, 635.

5 L. Wang, J. Zhang, N. Wang, X. Yang, Q. Wang and X. Yu, *Lett. Org. Chem.*, 2009, **6**, 392.

6 C. Wei, L. Ren and N. Gao, *Int. J. Biol. Macromol.*, 2013, **57**, 1.

7 H. Bertrand, D. Monchaud, A. De Cian, R. Guillot, J.-L. Mergny and M.-P. Teulade-Fichou, *Org. Biomol. Chem.*, 2007, **5**, 2555.

8 N. S. Oltra and G. Roelfes, *Chem. Commun.*, 2008, 6039.

9 C. H. Wang, G. Q. Jia, J. Zhou, Y. H. Li, Y. Liu, S. M. Lu and C. Li, *Angew. Chem. Int. Ed.*, 2012, **51**, 9352.
